# Developmental transcriptomics throughout the embryonic developmental process of *Rhipicephalus turanicus* reveals stage-specific gene expression profiles

**DOI:** 10.1186/s13071-022-05214-w

**Published:** 2022-03-15

**Authors:** Zhang Ruiling, Liu Wenjuan, Zhang Kexin, Wang Xuejun, Zhang Zhong

**Affiliations:** 1 Collaborative Innovation Center for the Origin and Control of Emerging Infectious Diseases, Shandong First Medical University (Shandong Academy of Medical Sciences), Tai’an, China; 2 School of Basic Medical Sciences, Shandong First Medical University (Shandong Academy of Medical Sciences), Tai’an, China; 3 Shandong Provincial Center for Disease Control and Prevention, Jinan, China

**Keywords:** Tick, Embryonic development, Transcriptome, Stage-specific

## Abstract

**Background:**

Ticks are important vectors and transmit diverse pathogens, including protozoa, viruses, and bacteria. Tick-borne diseases can cause damage to both human health and the livestock industries. The control and prevention of ticks and tick-borne diseases has relied heavily on acaricides.

**Methods:**

In the present study, using a high-throughput RNA sequencing (RNA-Seq) technique, we performed a comprehensive time-series transcriptomic analysis throughout the embryogenesis period of *Rhipicephalus turanicus*.

**Results:**

Altogether, 127,157 unigenes were assembled and clustered. Gene expression differences among the embryonic stages demonstrated that the most differentially expressed genes (DEGs) were observed in the comparisons of early embryonic stages (RTE5 vs. RTE10, 9726 genes), and there were far fewer DEGs in later stages (RTE25 vs. RTE30, 2751 genes). Furthermore, 16 distinct gene modules were identified according to weighted gene co-expression network analysis (WGCNA), and genes in different modules displayed stage-specific characteristics. Gene Ontology (GO) annotations and Kyoto Encyclopedia of Genes and Genomes (KEGG) pathway enrichment suggested that some genes involved in organ and tissue formation were significantly upregulated in the early embryonic developmental stages, whereas metabolism-related pathways were more enriched in the later embryonic developmental stages.

**Conclusions:**

These transcriptome studies revealed gene expression profiles at different stages of embryonic development, which would be useful for interrupting the embryonic development of ticks and disrupting the transmission of tick-borne diseases.

**Graphical Abstract:**

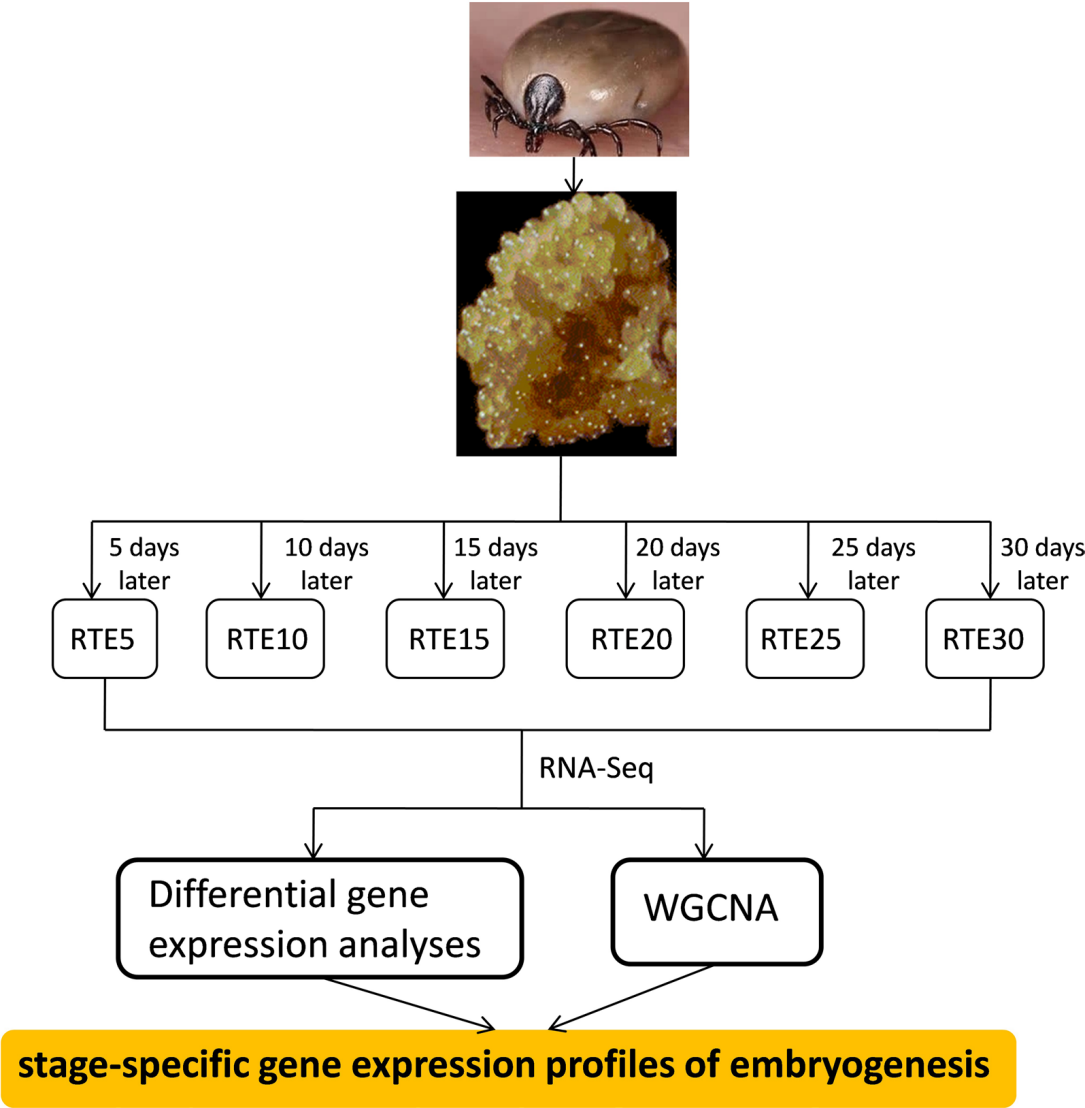

**Supplementary Information:**

The online version contains supplementary material available at 10.1186/s13071-022-05214-w.

## Background

Ticks (Acari: Ixodidae) are obligate ectoparasitic arthropods widely distributed across the world. The life-cycle of hard ticks typically includes eggs, larvae, nymphs, and adults (male and female). Blood meals are needed for survival, growth to the next developmental stage, and for reproduction. Then, engorged larvae and nymphs molt into nymphs and adults, respectively. Blood-feeding is a prerequisite for oviposition of adult females, and thousands of eggs can be laid by a single female tick [1]. Bloodsucking of ticks not only harms hosts by causing tick paralysis, allergic reactions, damage to the skin, decreased productivity, and decreased immune function, but also transmits disease-causing pathogens, such as protozoa, viruses, and bacteria, from the infected hosts to other hosts in subsequent blood-feeding [2–9]. Ticks are currently considered to be second only to mosquitoes as vectors of infectious diseases to humans and animals [10].

Ticks have few natural enemies, and vaccines against tick-borne diseases remain unavailable. The control of ticks and tick-borne diseases has largely relied on chemical insecticides. However, long-term and intensive use of insecticides has resulted in the increased resistance of ticks, and chemical residues of insecticides also contaminate the environment and agricultural products [11–15]. Therefore, excessive reliance on pesticides for tick and tick-borne disease control has been proven unsustainable [3, 10, 16–18]. The increasing resistance of ticks to these agents and the environmental pollution problems caused by acaricides had led to urgent requirements for novel sustainable methods that will greatly improve both current and future ticks and tick-borne disease control strategies.

Interrupting the life-cycle of ticks might be a feasible strategy to control ticks and block the transmission of tick-borne diseases [19]. Among all of the developmental stages of hard ticks, the embryonic development (from the eggs being laid to the larvae hatching) phase would be an optimal choice. As this stage exists off-host and will last for approximately 30 days, blocking strategies targeting the eggs will not affect their hosts. Additionally, embryonic development is vital for the whole life-cycle of ticks and involves an exclusive sequence of cellular and molecular processes that are not present in other stages [19]. Therefore, the identification of crucial molecular targets for the interruption of embryonic development would contribute to reducing the size of tick populations and would be helpful for preventing the transmission of both transstadial (stage-to-stage, also called horizontal) and transovarial (female-to-egg, also called vertical) tick-borne pathogens.

*Rhipicephalus turanicus* is widely distributed in Africa, Asia, and the Mediterranean regions [20]. Hosts of this tick species include goats, dogs, cattle, sheep, lions, and humans [20, 21]. This tick species has been implicated as a vector of several human and veterinary pathogens, such as *Rickettsia* spp. [22, 23], Crimean–Congo hemorrhagic fever virus, West Nile virus, *Babesia*, *Theileria*, *Anaplasma*, and *Hepatozoon* [20, 24–27]. Due to the lack of genomic resources and systematic research, the molecular mechanisms of the embryonic development of *R*. *turanicus* remain largely unknown. In this study, based on a high-throughput RNA sequencing (RNA-Seq) technique, we performed comprehensive time-series transcriptomic analysis of eggs of *R. turanicus* to provide global insights into the dynamics of gene expression during the successive stages of embryonic development and to identify development-related genes across different stages.

## Methods

### Tick collection and rearing

*Rhipicephalus turanicus* specimens were collected from grasslands in Tai’an, Shandong Province, and have been maintained in the laboratory since 2018 (Additional file 1). Ticks were fed on the ears of domestic rabbits for blood meals and were reared in an incubator at 70–80% relative humidity (RH) under a 12/12 h light/dark (L/D) photoperiod at 25 ± 1 °C after they were detached from their hosts. The eggs used in this study were collected from engorged female ticks (descendant of the same female *R*. *turanicus*) that fed on the same rabbit. Engorged females were examined daily to ensure that eggs could be collected in time. Laid eggs were collected every day from the start of oviposition and were kept in different plastic tubes covered with cotton mesh (to reduce water loss from evaporation and maintain surface gas exchange). The newly collected eggs were marked as 1 day, eggs of 5 days (RTE5), 10 days (RTE10), 15 days (RTE15), 20 days (RTE20), 25 days (RTE25), and 30 days (RTE30) were chosen, and approximately 300 mg of eggs (in three biological replicates) for each day was used for transcriptome analyses.

### RNA extraction, library construction, and sequencing

Total RNA was extracted using the RNAiso Plus reagent (Takara, Japan). RNA degradation and contamination were checked with denaturing agarose gel electrophoresis and RNA integrity was assessed using the RNA 6000 Nano assay kit of the Agilent Bioanalyzer 2100 system (Agilent Technologies, CA, USA).

Altogether 18 transcriptome sequencing libraries from six groups (with three biological replicates for each group) of eggs were constructed using a NEBNext^®^ Ultra™ RNA Library Prep Kit for Illumina (NEB, USA) according to the manufacturer’s instructions. High-throughput transcriptome sequencing was performed on an Illumina HiSeq platform, and paired-end reads (raw reads) with a length of 150 base pairs (bp) were generated.

### RNA-Seq data analysis

The quality and number of reads of each sample were assessed using FastQC version 0.11.4 [28]. Raw reads in FASTQ format were trimmed using Trimmomatic version 0.38 [29]. Clean data (clean reads) were obtained by removing adapter, poly-N and low-quality reads from the raw data. De novo transcriptome assembly was carried out using Trinity [30]. The Benchmarking Universal Single-Copy Orthologs (BUSCO) v3.0.2 program was utilized to assess the quality and annotation completeness of the genome assembly [31]. Then assembled unigenes were annotated by searching against the NCBI non-redundant protein sequences (Nr) and the Swiss-Prot database using BLAST alignment with an E-value cutoff of 1e−5. Gene functions were annotated by searching against the Pfam database [32] using the HMMER3 program [33]. Functional annotation by Gene Ontology (GO) terms was achieved by Blast2GO software (version 2.3.5, https://www.blast2go.com/) [34]. The unigenes were also aligned with the Cluster of Orthologous Groups (COG) database to classify and predict functions [35]. The best hits were used to determine the sequence orientations and coding sequences (CDSs) of the unigenes. Kyoto Encyclopedia of Genes and Genomes (KEGG) pathway analysis was performed using the KEGG Automatic Annotation Server (KAAS) [36].

### Differential gene expression analyses

The number of fragments per kilobase of the transcript sequence per million mapped reads (FPKM) was used to estimate the relative expression levels of unigenes. Differential expression analysis between groups was performed using the DESeq2 R package (version 1.20.0) with default normalization. *P*-values of the results were adjusted using the Benjamini and Hochberg approach for controlling the false discovery rate. Besides the DESeq2 package, significant differentially expressed genes (DEGs) between two compared groups were also determined using the edgeR package (version 3.12.1) with a fold change threshold of 2 and a false discovery rate (FDR) threshold of 0.05. Genes with an adjusted *P*-value < 0.05 were assigned as DEGs. Results from both packages were used for DEGs analyses to ensure the reliability of DEG assignment. DEGs of all six groups were subjected to principal component analysis (PCA).

### Weighted gene co-expression network analysis (WGCNA)

WGCNA (v1.47) was used to define co-expressed modules and hub genes. Based on pairwise correlations between genes, different modules can be divided into genes with similar expression profiles grouped into the same module [37, 38]. Each module was summarized by a single representative expression profile, which was referred to as the module eigengene. A hierarchical clustering tree was used for module classification according to the dynamic tree cut method. Then, modules with similar expression profiles were merged as merged dynamics. Gene connectivity was represented by edge weight and defined as the sum of weights across all edges of a node in the gene co-expression network analysis. Hub genes were defined based on the intra-modular connectivity which was identified by the CytoHubba plugin in Cytoscape v3.7.2.

### Quantitative real-time PCR (qRT-PCR) validation

To validate the RNA-Seq data, the expression levels of eight DEGs involved in embryonic developmental processes were randomly chosen and confirmed by qRT-PCR. Specific primers for these selected genes were designed by Primer Premier 6.0 (Premier Biosoft International, CA, USA) and are listed in Additional file 10: Table S1. Total RNA was extracted from the same batch of samples as those used in RNA-Seq, and reverse transcription was performed using a PrimeScript RT Reagent Kit with gDNA Eraser (TaKaRa, Dalian, China). RT-PCR was performed on an ABI 7500 system (ABI, CA, USA). Individual reactions were prepared with 100 ng of complementary DNA (cDNA) and a ChamQ SYBR qPCR Master Mix Kit (Cat#Q311-02, Vazyme, Nanjing, China) in a final volume of 20 μl. qRT-PCR reactions were carried out for 15 min at 37 °C, followed by 5 s at 85 °C, then followed by 40 cycles of two-step PCR for 10 s at 95 °C and 30 s at 60 °C. All reactions were carried out in triplicate for each sample. β-actin was used as an endogenous control to normalize expression levels. Cycle threshold (Ct) values were normalized using the 2^−ΔΔCt^ method [39], and one-way analysis of variance (ANOVA) was adopted to verify the significant difference (*P* ≤ 0.05).

## Results

### Sequencing, assembly, and annotation of the *R. turanicus* transcriptome

After quality trimming and filtering, 37,230,938 to 61,332,076 clean reads were obtained for 18 different libraries, with Q30 > 94% for all samples (Additional file 11: Table S2). Combined assembly based on total reads from all samples generated 127,157 unigenes and the N50 was 2356 bp. The average length of the unigenes was 1044 bp, and the shortest and longest sequences were 201 bp and 28,091 bp, respectively. For these unigenes, 33,151 were annotated, of which 33,107 (26%), 23,674 (18.6%), 13,354 (10.5%) and 13,793 (10.8%) unigenes were successfully annotated against known genes in the NR, KEGG, KOG, and SwissProt databases, respectively. A large number of mapped unigenes were matched with *Ixodes scapularis* (Additional file 2: Figure S1). BUSCO revealed 94.2% complete, 76% complete and single copy, 18.2% complete and duplicated, 3.9% fragmented, and 1.9% missing BUSCOs.

### Unigene functional classification

Functional classification based on GO demonstrated that annotated unigenes were divided into 58,986 GO terms and classified into 58 groups in three major GO categories (Additional file 3: Figure S2). The cellular process (7443 unigenes) was predominant in the biological process ontology, followed by metabolic processes (6879 unigenes) and single-organism processes (6553 unigenes). In the cellular component ontology, cell (4611 unigenes) and cell part (4,611 unigenes) exhibited the same abundance. Catalytic activity (6536 unigenes) and binding (5152 unigenes) were dominant in the molecular function ontology. All unigenes obtained from COG annotation (13,354 unigenes) were classified into 25 families (Additional file 4: Figure S3). Signal transduction mechanisms (2246 unigenes) were the most enriched family, followed by posttranslational modification, protein turnover, chaperones (1266 unigenes), and transcription (954 unigenes). Then, all assembled unigenes were mapped to the KEGG database to identify pathways in which genes were involved. All 23,674 unigenes were aligned to 145 pathways, and the most representative pathways were metabolic pathways (159 unigenes), lysosome (322 unigenes), and RNA transport (229 unigenes) (Additional file 12: Table S3).

### Comparison of gene expression profiles among different groups

**Fig. 1:**
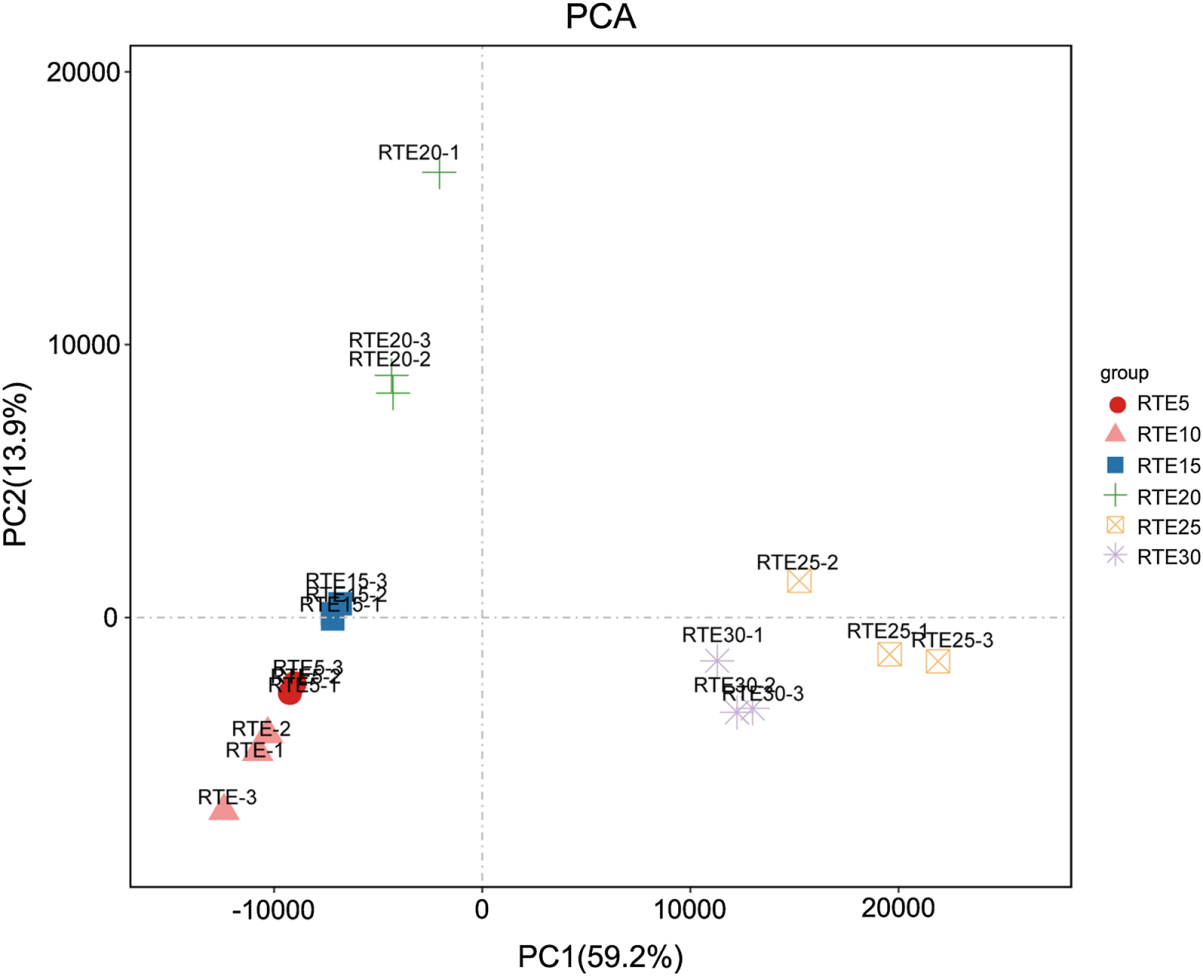
Principal component analysis (PCA) plot displays distance for transcripts of different samples

Based on the PCA results, the samples were differentiated according to embryonic development status (Fig. 1). Groups 1, 2, and 3 clustered together, group 4 clustered separately, and groups 5 and Group 6 had more similar gene expression patterns than those of other groups.

**Fig. 2:**
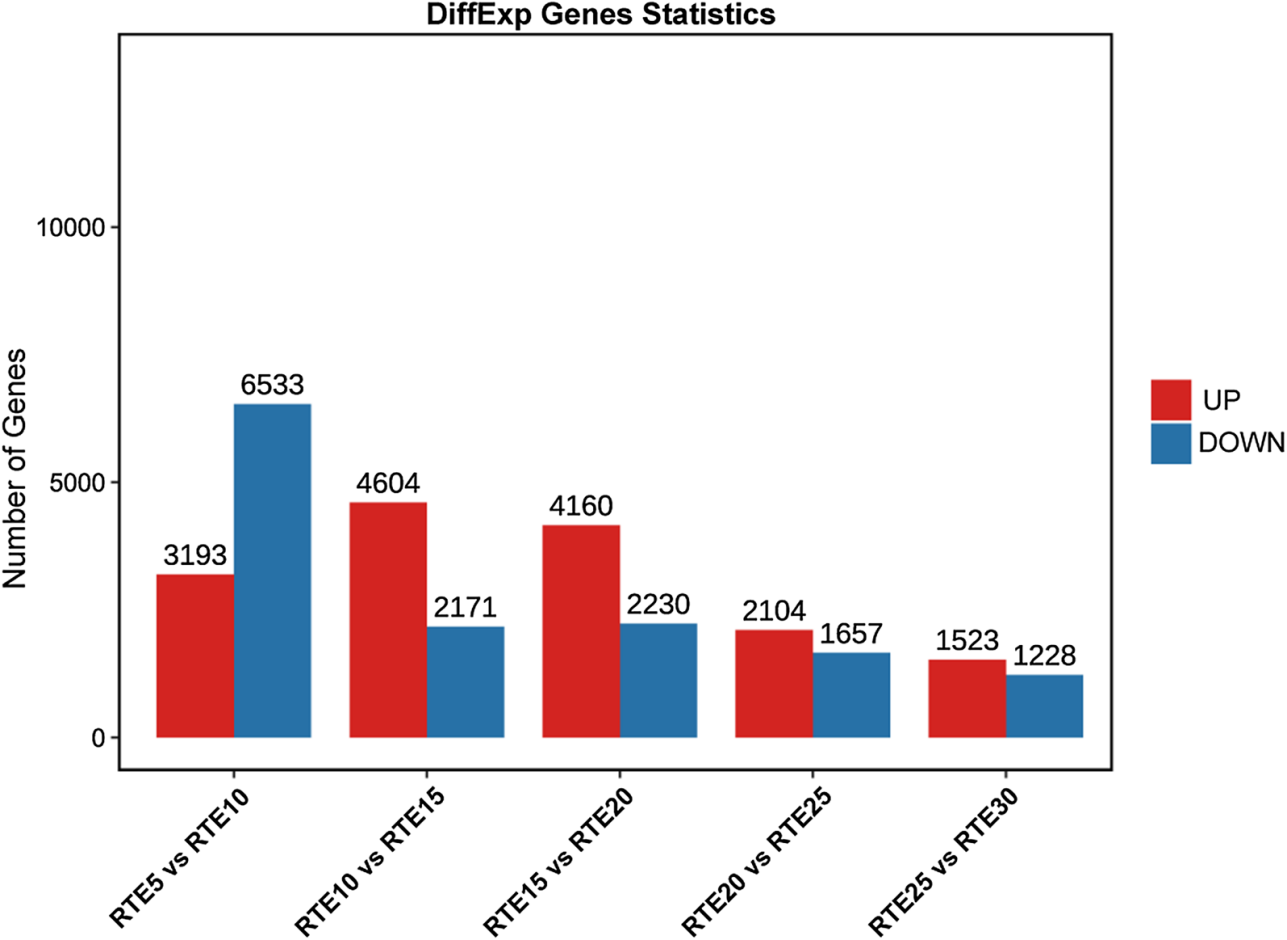
Numbers of DEGs between different embryonic developmental stages of *R. turanicus*. Red bars represent upregulated and blue bars represent downregulated genes. The *x*-axis indicates comparison groups; the *y*-axis denotes the number of genes

**Fig. 3:**
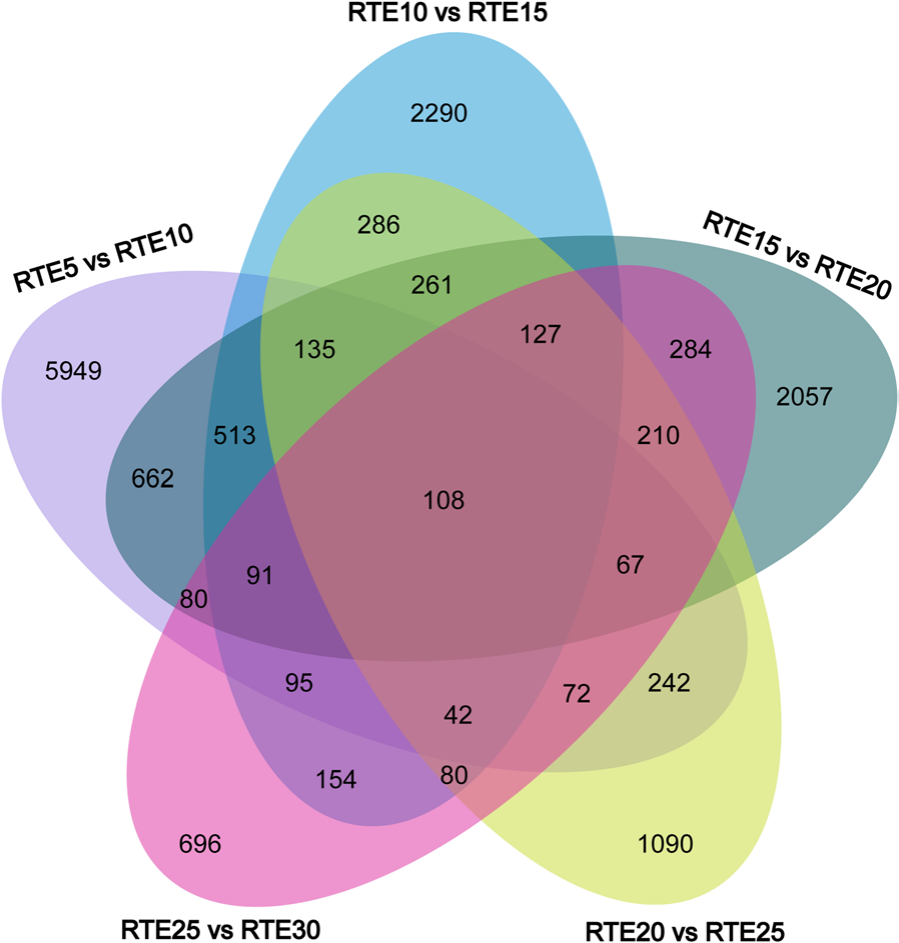
Venn diagram showing the number of DEGs expressed in different comparison groups

The FPKM method was used to assess the expression level and normalize unigene in each sample. Only genes that were differentially expressed in both edgeR and DESeq2 packages were selected for further analysis. DEGs between every two consecutive groups were identified. According to the comparative analysis, more genes were upregulated than downregulated in RTE10 vs. RTE15 (4,601/2,171 up-/downregulated), RTE15 vs. RTE20 (4,160/2,230 up-/downregulated), RTE20 vs. RTE25 (2,104/1,657 up-/downregulated), and RTE25 vs. RTE30 (1,523/1,228 up-/downregulated); while downregulated genes substantially exceeded upregulated genes in RTE5 vs. RTE10 (3,193/6,533 up-/downregulated) (Fig. 2). Comparisons between every two consecutive groups suggested that most of the unique DEGs were found in the comparison between RTE5 and RTE10 (5,949 genes), and only 696 unique DEGs were found between RTE25 and RTE30 (Fig. 3). Taken together, 108 DEGs were shared by all five comparisons of six time points.

GO enrichment analysis was carried out to investigate the functions of annotated DEGs. Among the annotated DEGs, the biological processes category contained the most GO annotations (7651 genes), followed by molecular function (7497 genes) and cellular components (4944 genes) (Additional file 13: Table S4). Enriched biological processes of RTE5/RTE10 were generally associated with DNA binding (GO: 0003677), nucleic acid binding transcription factor activity (GO: 0001071), and regulation of transcription from the RNA polymerase II promoter (GO: 0006357). Enriched biological processes of RTE10/RTE15 were DNA binding (GO: 0003677), gated channel activity (GO: 0022836), and ligand-gated ion channel activity (GO: 0015276). Receptor activity (GO: 0004872), transmembrane receptor activity (GO: 0099600), and molecular transducer activity (GO: 0060089) were the dominant biological processes in RTE15/RTE20. Fatty acid metabolic process (GO: 0006631) was the most significantly enriched GO term in both RTE20/RTE25 and RTE25/RTE30 (Additional file 13: Table S5).

The functional classifications and predictions were further analyzed by searching DEGs against the KEGG database to determine the involvement of biological pathways in the embryogenesis of *R. turanicus*. There were 140 KEGG pathways were predicted in RTE5/RTE10 and RTE10/RTE15, respectively (Additional file 14: Table S6). The most enriched pathways of RTE5/RTE10 included axon regeneration (ko04361), Wnt signaling pathway (ko04310), and MAPK signaling pathway-fly (ko04013) (Additional file 5: Figure S4; Additional file 14: Table S6). DNA replication (ko03030), neuroactive ligand–receptor interaction (ko04080), and pyrimidine metabolism (ko00240) were highly enriched in RTE10/RTE15 (140 pathways). The representative pathway in RTE15/RTE20 (136 pathways) was neuroactive ligand–receptor interaction (ko04080). Biosynthesis of unsaturated fatty acids (ko01040), fatty acid elongation (ko00062), and fatty acid metabolism (ko01212) were the top three highly enriched pathways in both RTE20/RTE25 (126 pathways) and RTE25/RTE30 (119 pathways). Two drug metabolism pathways were involved, including drug metabolism-other enzymes (ko00983) in RTE5/RTE10 and RTE15/RTE20 and drug metabolism-cytochrome P450 (ko00982) enrichment in RTE10/RTE15, RTE20/RTE25, and RTE25/RTE30 (Additional file 5: Figure S4; Additional file 14: Table S6).

### Correlation of differentially expressed genes and WGCNA

WGCNA identified modules of co-expressed genes and candidate hub genes for each time point. After modules with similar expression profiles were merged, 16 distinct gene modules were identified (Additional file 6: Figure S5). The number of genes in each module were 4748 (blue), 3276 (brown), 3086 (green), 2255 (darkgrey), 1154 (magenta), 697 (darkgreen), 390 (darkred), 369 (tan), 296 (lightyellow), 244 (midnightblue), 231 (lightcyan), 185 (royalblue), 122 (orange), 114 (white), and 57 (steelblue). Unassigned genes (22 genes) were placed into the “grey” module (Additional file 7: Figure S6).

**Fig. 4:**
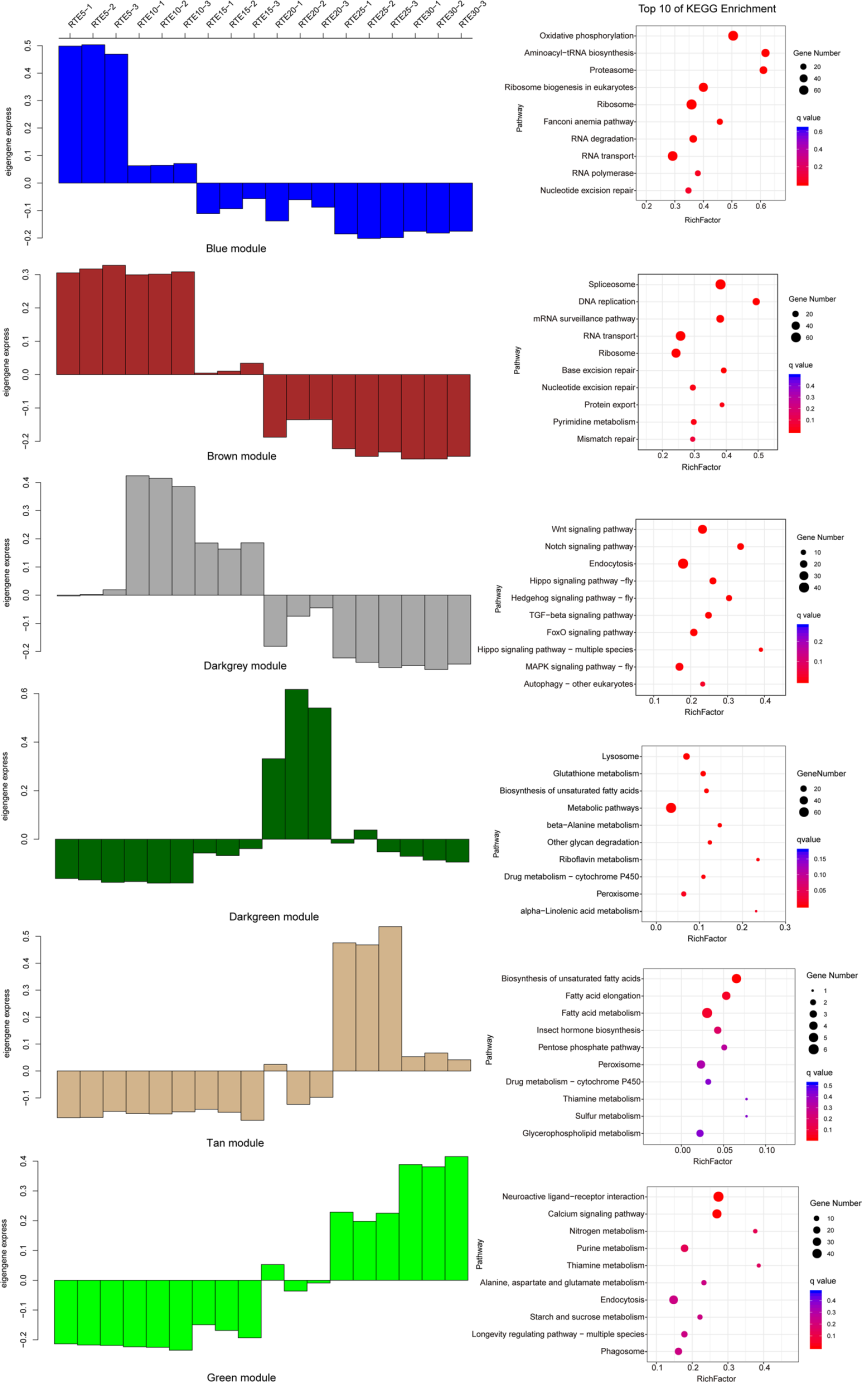
Expression pattern (histogram) and corresponding KEGG enriched pathway (scatter plot) analysis of blue, brown, darkgrey, darkgreen, green, and tan modules. In the histogram, the *x*-axis represents the sample, the *y*-axis represents the expression profile of the eigengene; the *x*-axis and *y*-axis in the scatter plot are the rich factor and KEGG pathways. Rich factor refers to the ratio of the number of genes located in the KEGG pathway and the total number of genes in the KEGG pathway. The larger the rich factor, the greater enrichment.

The genes in the module blue, brown, and darkgrey were upregulated in the early stages of embryogenesis (RTE5, RTE10), the lightcyan module consisted of genes upregulated in RTE10 and RTE20 of embryogenesis, while genes in the green and tan module were merely upregulated in RTE25 and RTE30 (Fig. 4). The upregulated expression patterns of the darkred, lightyellow, and royalblue modules were maintained in both RTE15 and RTE20. Additionally, the genes of the orange and steelblue modules were upregulated in RTE15 and RTE30, respectively. The genes of the darkgreen module were mainly upregulated in RTE20, while the genes of the white module were upregulated in RTE5 and RTE30, respectively. The magenta module genes were upregulated in RTE10 and RTE15, whereas the midnightblue genes were upregulated in RTE20 and RTE25 (Additional file 8: Figure S7).

GO analysis and KEGG pathway enrichment were performed to explore potential biological processes associated with different embryogenic developmental stages. GO term enrichment analysis revealed that the genes of the blue, brown, darkgrey, darkgreen, tan, and green modules were related to three main categories (biological processes, molecular function, cellular components). The most enriched GO subcategories of these six modules were cellular process, metabolic process, single-organism process, cell, cell apart, catalytic activity, and binding, respectively (Additional file 9: Figure S8).

The most enriched KEGG pathways in the blue module included oxidative phosphorylation (ko00190), aminoacyl–transfer RNA (tRNA) biosynthesis (ko00970), and proteasome (ko03050). Spliceosome (ko03040), DNA replication (ko03030), and messenger RNA (mRNA) surveillance (ko03015) were the top three pathways in the brown module. The Wnt signaling pathway (ko04310), Notch signaling pathway (ko04330), and endocytosis (ko04144) were abundant in the darkgrey module. In the darkgreen module, lysosome (ko04142), glutathione metabolism (ko00480), biosynthesis of unsaturated fatty acids (ko01040), and peroxisome (ko04146) were significantly enriched. Biosynthesis of unsaturated fatty acids (ko01040), fatty acid elongation (ko00062), and fatty acid metabolism (ko01212) were predominant in the tan module. The green module was enriched with neuroactive ligand–receptor interaction (ko04080), calcium signaling pathway (ko04020), and nitrogen metabolism (ko00910) (Fig. 4).

**Fig. 5:**
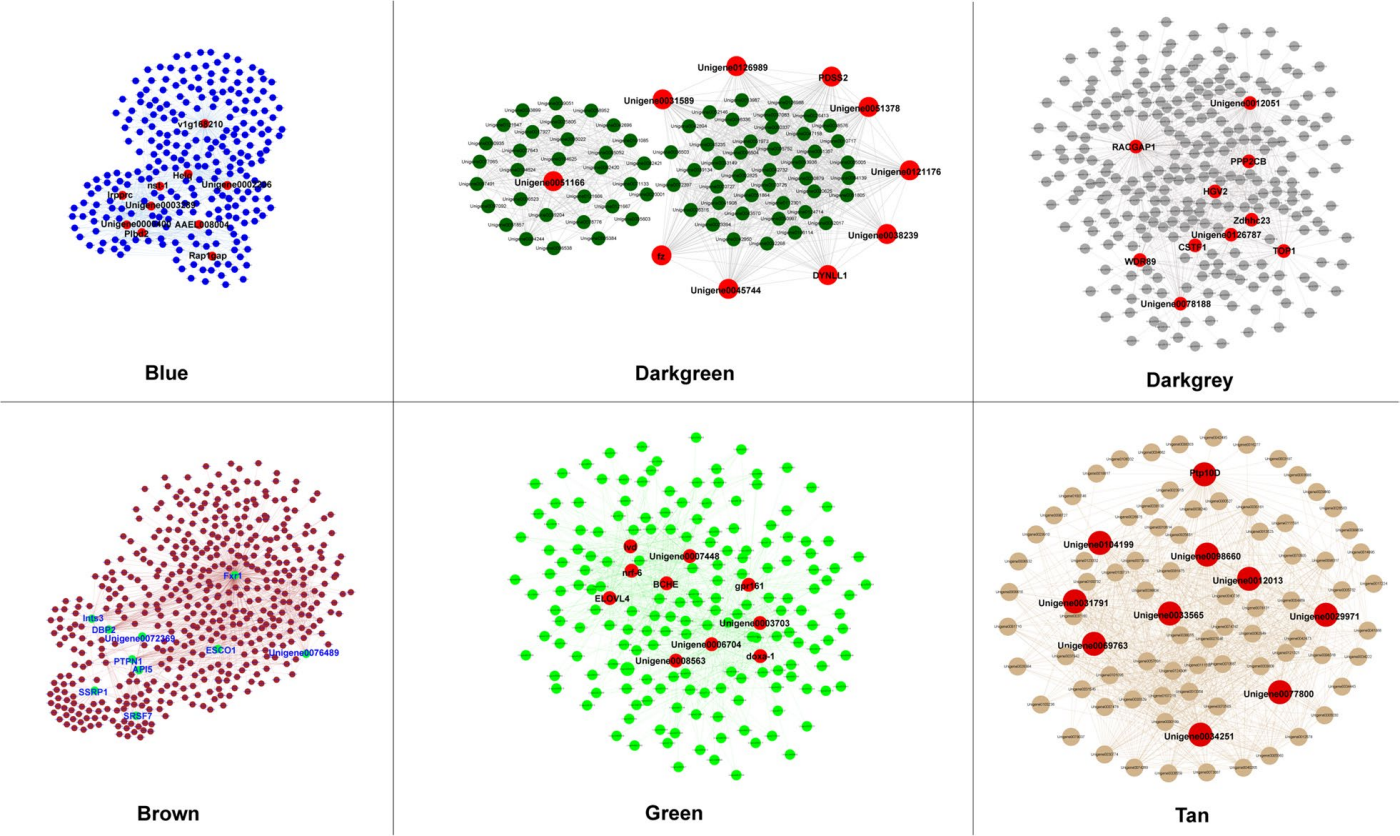
Networks of genes in different modules. The top ten hub genes are shown in red in the blue, darkgrey, darkgreen, green, and tan modules; the top ten hub genes are shown in green in the brown module

Networks were constructed to explore relationships among genes, in which each node represents a gene and the edges between genes represent co-expression correlations. To capture a more meaningful correlation, genes with the highest connectivity were selected as the hub genes in each module (Fig. 5). The hub genes of the blue, brown, darkgrey, and tan modules were translation initiation factor 3 subunit (v1g168210), synaptic functional regulator FMR1 (Fxr1), rac GTPase-activating protein 1-like (RACGAP1), and tyrosine-protein phosphatase 10D (Ptp10D), respectively. However, the hub genes of the green and darkgreen modules are still unannotated (Additional file 15: Table S7).

### Quantitative real-time PCR analysis

**Fig. 6:**
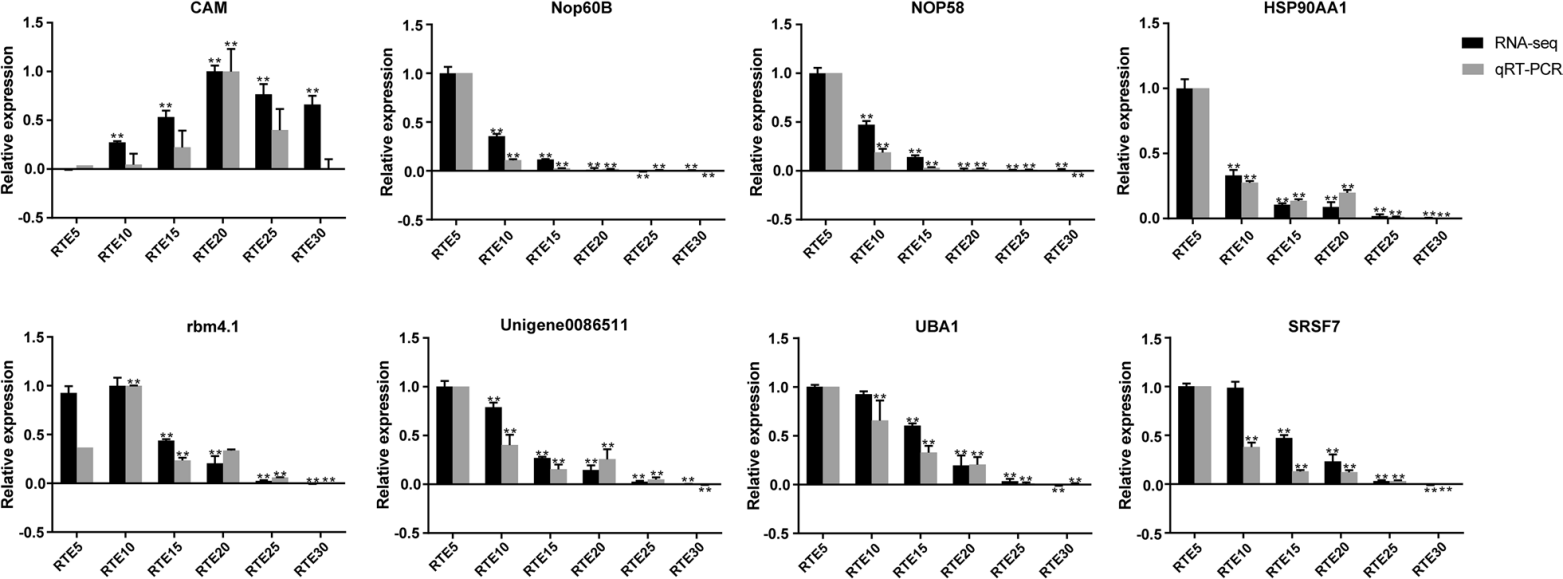
Comparison of expression values of eight genes by RNA-Seq and real-time RT-PCR analyses. The *x*-axis represents the developmental time point; the *y*-axis represents the relative expression levels. Asterisks indicate statistical significance at the level of *P* < 0.01

The expression profiles of 8 randomly selected genes (Nop60B, unigen0086511, SRSF7, HSP90AA1, NOP58, rbm4.1, UBA1, CAM) were quantified using qRT-PCR to validate the accuracy and reproducibility of the expression profiles of RNA-Seq. The results from the qRT-PCR experiments were consistent with those observed in transcriptome analysis, and both of the strategies revealed similar trends in the upregulated or downregulated genes (Fig. 6).

## Discussion

Blood-sucking arthropods are responsible for major epidemics throughout the world, causing thousands of deaths and great harm to public health. Ticks affect both humans and animals, causing considerable losses to the cattle industry worldwide and incurring substantial damage to livestock [40]. At each blood meal, ticks can become integrated into the epidemiological chain of pathogen transmission by means of so-called transstadial or transovarial passages.

The cattle tick *R*. *turanicus* is an obligate hematophagous arthropod, and embryogenesis is one of the most important life stages, as it involves large changes to the RNA transcript profile [41]. Identifying transcriptional changes during embryogenesis is of crucial importance for unraveling the evolutionary, molecular, and cellular mechanisms that underpin patterning and morphogenesis [42]. Most of the knowledge about the developmental gene regulation of Arthropoda has been derived from the *Drosophila* paradigm [43]. However, the involvement of genes in specific developmental processes is usually influenced by many factors, including species and the expression patterns of genes. Developmental transcriptomics across all embryonic stages can provide gene expression divergence information spanning different developmental time points and offer novel insights into the genetics of embryogenesis.

Numerous efforts have been made in basic and applied tick and tick-borne disease control research [18, 44–47]. However, as one of the important stages of the tick life-cycle, research on embryos is limited, and available studies mainly focus on the morphological, histological, and ultrastructural characteristics of embryonic development [48, 49]. This paucity of early-stage information hinders the implementation of targeted approaches, such as RNA interference or characterization of vaccine candidates [50, 51]. Our study is among the first to provide a comprehensive and biologically relevant catalog of transcripts for future research aiming at controlling the population of ticks in the early stages of their development [52].

In the present study, the transcriptomes of six different developmental points that were determined according to the days after oviposition and covering the entire embryonic developmental process of *R. turanicus* were compared. PCA demonstrated that a few individuals from the same developmental stage were not closely clustered (Fig. 1), indicating that there were variations among individuals from the same embryonic stage [41]. Close clustering of Group 1, 2, and 3 indicated that the gene expression profiles were very similar during the early stages (RTE5 to RTE15) of embryogenesis, whereas the divergence of Group 4 from all other groups suggested that great changes in the gene expression patterns occurred at this time point (Fig. 1). According to the variations in gene expression, the changes between RTE5 and RTE10 were more intense than those between RTE25 and RTE30 (Fig. 2), confirming that more dramatic changes occurred during the earlier embryonic developmental periods.

Embryogenesis is a complex process involving an elaborate network of signaling pathways. Comparisons of gene expression among different stages revealed that a relatively high proportion of DEGs were present during early embryonic development stages, suggesting that more intense changes occur during the earlier developmental periods. The GO term and KEGG enrichment pathway analyses showed that the annotated DEGs participated in multiple physiological processes and that the expression levels of fundamental development-related genes were high during the early embryonic developmental stages (Additional file 3: Figure S2; Additional file 5: Figure S4; Additional File 15: Table S6). However, DESeq pairwise differential expression analysis might miss some expression profiles using only pairwise comparisons. WGCNA classifies the whole transcriptome into different expression patterns based on the comparison of all genes in all samples, which can help to obtain a whole picture of the expression profiles of embryogenesis. Genes involved in the same biological process were highly correlated among samples, and co-expressed genes throughout different embryonic developmental stages of *R. turanicus* clustered into 16 modules (Additional file 6: Figure S5), which provided more detailed gene expression dynamics and enrichment of pathway profiles according to embryonic developmental stages.

Limited overlap of these genes across embryonic developmental stages was observed, and this is consistent with previous phenotypic reports [48]. Stage-specific transcript characteristics were evidenced by the functional categories of the modules to which they belonged (Fig. 4). The main biological processes (e.g., RNA transport, DNA replication, and mRNA surveillance) in which the genes were upregulated in the blue and brown modules indicated that cell division is particularly intense during RTE5 and RTE10. These results are consistent with the results of morphogenetic processes, which suggested that intense cell proliferation occurred during the early stages of tick embryonic development [48]. Moreover, the significant enrichment of aminoacyl–tRNA biosynthesis, ribosome, and spliceosome gave a signature of intense protein production that could be related to secretory cells. Therefore, we can deduce that salivary glands might be formed in this stage.

The Wnt signaling pathway regulates a huge variety of embryonic developmental processes by inducing transcriptional or morphological changes in responding cells [53, 54]. Embryonic development analysis of *Rhipicephalus microplus* confirmed the expression of the Wnt signaling pathway during early embryonic development [48]. The enriched Wnt signaling pathway in the darkgrey module in this study indicated that the RTE10 and RTE15 stages may correspond to stages 4 and 5 of *R. microplus*, in which cumulus cells and axis establishment were detected. In addition, fundamental developmental genes related to the Notch [55], Hippo [56], Hedgehog [57, 58], transforming growth factor beta [59–61], and mitogen-activated protein kinase (MAPK) [62] signaling pathways showed higher expression during RTE10 and RTE15 stages than others (Fig. 4). Therefore, these stages are characterized by more active biological processes, including organ and tissue formation.

Lysosomes are membrane-delimited organelles that contain strongly hydrolytic enzymes and serve as the main digestive compartment of cells [63, 64]. Peroxisomes are essential organelles of eukaryotic cells that play a key role in the oxidation of fatty acids and the generation and removal of hydrogen peroxide [65]. Both lysosomes and peroxisomes are immune-related pathways, and the high enrichment of these pathways in the darkgreen module suggested that *R. turanicus* embryos have a relatively complete immune system at RTE20. Moreover, the high expression of immune-related pathways also indicated that immune defense is vital to this embryonic developmental stage. The embryogenesis of the tick is typically described as an energy-consuming process [40]. Energy metabolism is essential to supporting the molecular backbone needed for cell proliferation, differentiation, and embryonic growth. The catabolism of biomolecules, such as carbohydrates, is the main source of energy for the developing embryo [66]. These studies elucidated the highly enriched metabolism-related pathways during the later embryonic developmental stages (RTE25, RET30). Furthermore, the biosynthesis of unsaturated fatty acids and fatty acid metabolism, which are probably involved in exoskeleton structure formation (e.g., wax synthesis), were found in the later stages of embryonic development of ticks [48]. These results revealed that gene expression dynamics and patterns are highly varied among different embryonic developmental stages. However, highly enriched energy metabolism pathways, including oxidative phosphorylation in the blue module, sulfur metabolism in the tan module, and nitrogen metabolism in the green module, crossing multiple time points confirmed that embryogenesis of ticks is an energy-consuming process [40].

Additionally, drug metabolism-cytochrome P450 and drug metabolism-other enzymes maintained high expression during all stages (Fig. 4; Additional file 14: Table S6). Cytochrome P450s constitute a diverse and important gene superfamily in all organisms and are known to catalyze a diverse range of chemical reactions important for both developmental processes and the detoxification of exogenous compounds [67]. The overexpression of drug metabolism pathways suggests the high metabolic level of *R. turanicus* embryos to hazardous substances, while it is difficult to illustrate whether endogenous or exogenous substances induced the high expression levels of cytochrome P450 and related enzymes in the present study. Since all egg samples used in this study were kept in sterile plastic tubes and covered with cotton mesh, sterilized water was added onto the cotton mesh to keep the environment moist, as needed for the incubation of eggs. Additionally, ancestors of these eggs have been maintained in the laboratory since 2018, and they have not been in contact with any harmful substances during this time. Therefore, we speculate that endogenous products, which cannot be released in time due to the existence of wax in eggs, caused the high expression of drug metabolism pathways. Further research is needed to gain a better understanding of the role of drug metabolism pathways in the embryonic developmental stages of ticks and to identify possible new targets for the development of novel control measures against ticks.

Co-expression networks of each module were constructed and hub genes were identified accordingly (Fig. 5; Additional file 15: Table S7). However, as most of the candidate hub genes are functionally uncharacterized, it is currently impossible to correlate these hub genes with crucial biological functions in the embryonic developmental processes of *R*. *turanicus*. Directly linked genes usually have similar functions and may be involved in the same biological pathways related to embryonic development (Fig. 5; Additional file 15: Table S7). Therefore, the present research revealed new insights for further studies, as the hub genes may act as potential targets for further exploration of detailed biological functions to shed light on possibilities for the control of ticks and tick-borne diseases.

## Conclusion

In this study, we recapitulated the time-series transcriptomic profile of embryonic development of *R. turanicus*. The results demonstrated that not all genes are expressed at once, but each has its own specific temporal expression pattern. WGCNA classified all DEGs into 16 modules, and co-expression networks provided clue candidate hub genes of each module. KEGG enrichment pathways suggested that the most significant expression changes occur during the early embryonic developmental stages, particularly in RTE10 and RTE15, in which a large set of genes involved in organ and tissue formation are upregulated. In the later embryonic developmental stages, more genes were involved in metabolism-related pathways. These different expression profiles should be taken into consideration in the further exploration of target genes to interrupt the embryonic development of ticks.

## Supplementary Information

**Additional file 1**. The partial mitochondrial genome (9585 bp) used to identify species and the BLAST results against NCBI.

**Additional file 2: Figure S1**. Species distribution of Nr annotation results. The *x*-axis represents the name of species; the *y*-axis represents the number of unigenes annotated to different species.

**Additional file 3: Figure S2**. GO functional classification of unigenes. The *x*-axis represents the GO term; the *y*-axis represents the number of genes classified into each GO term.

**Additional file 4: Figure S3**. COG functional classification of unigenes. The *x*-axis represents the COG category; the *y*-axis represents the number of genes classified into each COG category.

**Additional file 5: Figure S4**. KEGG enriched pathway analysis of genes between adjacent embryonic developmental groups. The *x*-axis represents the rich factor, the *y*-axis represents KEGG pathways. Rich factor refers to the ratio of the number of genes located in the KEGG pathway and the total number of genes in the KEGG pathway. The larger the rich factor, the greater the enrichment.

**Additional file 6: Figure S5**. WGCNA of genes in different embryonic developmental stages. Hierarchical cluster tree showing co-expression modules identified by WGCNA. Each leaf on the tree represents one gene. All genes were grouped into 16 different color-coded modules according to their expression pattern.

**Additional file 7: Figure S6**. Gene numbers of different modules.

**Additional file 8: Figure S7**. Express pattern of lightcyan, darkred, magenta, lightyellow, orange, midnight, steelblue, royalblue, and white modules. The *x*-axis represents the sample; the *y*-axis represents the expression profile of the eigengene.

**Additional file 9: Figure S8**. GO functional classification of the blue, darkgreen, brown, tan, darkgrey, and green modules. The *x*-axis represents GO terms; the *y*-axis represents the number of genes classified into each GO term.

**Additional file 10: Table S1**. Primers used in qRT-PCR.

**Additional file 11: Table S2**. Summary of output statistics from different samples.

**Additional file 12: Table S3**. KEGG enriched pathways of all unigenes.

**Additional file 13: Table S4**. Gene numbers classified into three GO catalogs. **Table S5**. Top 20 GO terms of different comparison groups.

**Additional file 14: Table S6**. Enriched KEGG pathways of differentially expressed genes between adjacent embryonic developmental groups.

**Additional file 15: Table S7**. The top ten hub genes of the blue, brown, darkgrey, darkgreen, green, and tan modules.

## Data Availability

Raw RNA-Seq sequences were deposited in NCBI Sequence Read Archive (SRA) database with accession number: PRJNA741544.
