# Supplementary material for: Developmental transcriptomics throughout the embryonic developmental process of Rhipicephalus turanicus reveals stage-specific gene expression profiles
Source: Parasit Vectors. 2022 Mar 15;15:89. doi: 10.1186/s13071-022-05214-w (PMC8922761; doi:10.1186/s13071-022-05214-w)

**Dataset S1.** The partial mitochondrial genome (9585 bp) used to identify species and the blast result against NCBI.

TAACATAAAGGAATAATTAAAAACATTAAAAAATAAATAATTGTGGTGATTTTACTTATTAAATCAAAAGGGTATTCAATAGGTATTGCTCCAAGATATGTCAGTATAATAAAACAATTTACTAAAATTCAAAATATTAATTTAAATAAAATATTAAAGTAAAAAGATGATATTTTATTATTTATTGTGAAACAAAATCTCAAAATAATAACAATTGATATTACTAGAGCGATTACCCCTCCTAATTTATTAGGAATTGAACGTAAAATTGCATAAGCAAATAAAAAGTATCATTCTGGTTGGATATGAGGAGGGGTAATTATAGGGTTGGCAATATTAAAGTTTTCTGCGTCTATAAATCCGTAAGGATTGATAATAATAAAATAAGAAAAAATTATTAAAATTACAAAAATTCCTAATAAATCTTTAATGGTAAAATAAGGATGGAAAGGAATTTTATCTAAATTTATTGTTACACCGAGAGGATTTCTAGATCCTTTTTCGTGGATTAAGGTAATATGAACAACAGTTAATAACAATAAAATAAATGGTAATAAGAAATGTAAAGTGAAAAATCGAATTAAAGTATTATTATCAACTGAAAATCCTCCTCAAATTCAATAAGTAATTGATATTCCAATATATGGAATTGCTGAAATTAAGTTTGTAATTACGGTGGCTCCTCAAAAAGATATCTGTCCTCAAGGCAAAATATAACCTAAAAAAGCTGTAGCTATTAGCATAAAAATAATTAAAATTCCAGATATTCATACATTTTTAAAAAAAAATGAGGAATAATAAATTCCTCGAGCTATATGAATATAAATAAAAATAAAAAAAATCGATGCTCCATTAGCGTGAATTGAACGAATTAATCATCCATTATTTACATCCCGTTGAATATGAGAGATTATTGAAAAAGCTGTCGTAATATCACTCGAAAAGTTTATTGCTAGAAATAAACCTGTAATAATTTGGGTTAACAAGCAAATTCCTAATAAAGAACCAAAATTTCATATATAAGAAATATTTGAAGGAGTTGGTAAATTTTTTAAAGGATTAATTAGTTTTAACATAAGTTTAGTTAAACTTTTTAATAGGAGCATTAATTCATCTTAAATTTTTAGAAATAATAATTAATATAACAATTAAAAAGATTAATATTAAAATAATAATATTAACAAAGTTTATAGTATAAACTTTGTTAATATTAATTGTTTCTAATTTTGTTATAAATGTATTATATGGAATTAAAATCAATATTAAGGTAAAAGTGACTCTAAGTTTTTTATAAAATCTTATTTTTTTATTTGGACATAATCTAATTATATATATAAAAATAATTAATATTCCCCCTAAAATAATTAAAATTATTATTATTGATACAAAAGAAAACTGATAAATAAAATAAAATATTAAGGATAAGAATAATGTTAATAAAATTAATGAAGATAATATTATGATAGGATGTGAGTTATTATTAATGCTTTGTGTTTATTTTTGTTTATTTAATTTATTGGGGATTTTTGTTTTTTTGATTAGAATTGTCTGTGAAGCTGGTTTAGCTTTAAGATTATTAGTAATAATAAGATTTTATTATGGTAATGAATTAATAATAAGTATAAATTTAATTAAATGTTAACAATTCTTTTAATATCAATTTCAATTTTATGTTTATTTTTTTTAGTTAATCCTTATCAAATCATAATTTATCTTATAATTATAACTTCATTTTTACTTATTAAGGGTTTAATCAATAATGCCGATTTATTTATTAATTTTTTCTTTTTTGATTTAATAAGATTAAGTATGATTATTTTAACAGTTTGAATTTCATTACTTATGATTATAGCAAGAAAATTAAATAATAATTATAAAAATAAGACTTTTAATTTCTATTTATTATTAATAATGAATTTATTATTTATTTGTTTTTTAGCGGAAAATTTGTTAATATTTTATTTATTTTTTGAAGCAGTATTATTTCCTATTGTTTTAATAATTAGTGGTTGAGGGTCTCAACCAGAACGAATTCAAGCTGGATTTTATATATTAATTTACACAGTATTTGGCTCTCTTCCTTTATTAATCTTAATACTTTTAAAATATAAATCATTAAGAATTATTTTTAATGAATGATTATTTAATGAAATAGGGTTAATTTTTTTTTTCATGGTATTAGGTTTTTTAGTAAAAATTCCAATATTTTTATTCCATTTATGATTACCTAAAGCTCATGTTGAGGCCCCAATTGCTGGGTCAATAATTTTAGCTGGAGTCTTATTAAAACTAGGATTTTATGGATTATATCGATTCAAAAGTTTTTTTTTTTTAGATTTACTAAAATTTTCTTTTATGTTAATTATTATTTCTATATGAGGAGCAGTATTAATTAGTATTTATTGTCTATATCAATATGATATTAAATCACTAATTGCATATTCCTCAGTTTCTCATATAGGAATTACGTTAGCCGGATCTATTACATTTCAATTACATAGAAGATTTGGTATATTGATAATAATAATTGGTCATGGTTTATGTAGATCTGGTCTTTTTTGTTTAAGAAATATAATTTATGAACGATTACATACTCGAAGTATTTTAATAATTAAAGGAATAATTTTAATTTTTCCAAATTTAAGGTTATGATGATTTTTATTTAGAATCATTAATATATCAGCTCCAATAACCATAAATCTTTTTGGTGAACTATTTTTAGGAATAAGATTGATAAAATATAGATTATTATTGTCATTACCTTTAATAATAATAATTTTTCTCAGAGCTTGTTATTCTATGTATATGTATAGTTATATTAATCATGGTCAAAGTTGAATAATTTTTAGTAATAAAATAATCTCTCTTCGAGAATATTATTTAATATTTCTTCATATTTTTCCTATAATTATATGATTTTTTAAAATTAATTTTTTTATGAAATGAATTTAATTAAATAAAAAATTGAAAATGACCTTAAGAAAATTATTGCCTTTTCAACTCTTAGACAGTTATCAATTATAATATAACTCTATCATTACAATTAACTAATTTAGCATTTTTTCATTTAATTATTCACGCTATTTTTAAATCAATATTATTTTTATGTGCCGGTTTTATTATTCATAACTTAATAGGAAATCAAGATATTCGATTTTTATCTGATTTTTTTAAATTTAGTCCTTTAATTATAAGATGTATAGTAATCGGAATATTATCTTTAATGGGATTTCCTTTTATTGGAGGGTTTTACTCAAAAGATGTAATTATAGAATTTTTTTTTTTAATTAGAAAAAATTTTATAGAAATAATTCTATTTTTTATAGGAATTGCCTTTACTTTTCTTTATAATTTTCGACTTTTTTATATACTTTTATTAAAAGGAACATCATTTAATATTATAATAAAGAATAACTTAAATATTTTCATAAATTATCCAATTTTTAATTTAACAATATATTTATTAATTGTTAGAAATTTAATAAGATGATTATTATTACCAGTTAAATTATTTATTAAATTTAATTTAAAAATTTAAGTAAAAATGACGTTATTAGTAAAAGTAAAAAAATTTTTTTTTAATTAAATTAGAAACTTAATTATAGAACTTTAATTTAAAAGAATTAGGCAAATTAATTTTCTGACTGTTTAATAAAAACAACGCATTTAGATTATTAATAAATGCAAATCCTGCTCAATGAAACTTAAATTGCTGTAGTATTTTGACTATACAAAGGTATTGAAATAAGATTTTAATTGAATGCTAAGAGAATGGACTCTCAGAGAAAAACTTTTTTTAAATTAAAAATTGAAATTTTTTTAATTTGTGCAGAAACAATTATTAATATTAAGGACAAGAAGACCCTATGAATTTATTAAATTTCATTCAATATGTAATTACTATTGAAGAAATTTTGGCTGGGGCGGCTAAAAAATATTTTAAACTTTTTAAAAATAAAATGATCCATTATTAATGATTATATGTTAAATACTCTAGGGATAACAGCGTTATATTTTTTGATAGACCATATTGACAAAAAAGTTTGCGACCTCGATGTTGGATTAGGATACTTTTTTAATGCAGATATTAAAAAAAGAAGTTTGTTCAACTTTCAATTTCCTACATGATCTGAGTTTAGACCGATGAGAATCAGGTTGGATTCTATCTTAATAATAAAATAATTTTAATTAGTACGAAAGGAATTTTAAAATATAATTATTAGGATAAACAACTTTATTTGCATCAATTTTTGTAATTATTAAAGTGGCAGAACTTAATGCGAGGAATTTAAGCTTCCTCTATGAAAATTTCCTTTAATAATTTTAAATTTTATTTATTTTTTTATTTTAATTTTAATAGTGCTATTAAGAATTGCTTTTTTCACGTTAATAGAACGAAAATTTTTAGGATATTGCCATATTCGAAAAGGCCCAAATAAAACAGGAATATTAGGGCTTCTTCAGCCAATTAGTGATGCATTAAAACTTTTTAGAAAAGAAATAAATAAAATATTTTATATAAATAAGTTCATTCAAATTATTTCTCCACTTATTATAATCCTAATAATAATAATAATATGAATAATTTTTTATTTTTCAAATAATGCAATAAATTTAAATATAAGAATCATCTTTTTCCTTTGTATTTCAAGATTAGCAAGATACACAATTTTATTTAGAGGATGAGCCTCTAACTCAAAATATTCGTTAATTGGTTCTTATCGAGGATTTGCCCAAGTAATTTCTTATGAAGTAAGAATAGCCATAATTTTAATTTCACTAGCTATTATCCCTCAAAGATATAATTTTATTTCATTCCTTAAATTTCAAGAAACGTTTCCTTTAATTTTCAGATTTTTACCAATTTTTATTATTTGGATTATTACTATTTTAGCAGAATTGAATCGAGTACCATTCGATCTTGCCGAAGGAGAATCTGAGCTTGTTTCAGGGTTTAATATTGAGTATGGTTCTTGATTATTTGCAATTATCTTTATATCAGAATATGGGAATATTATAATAATTAGTTTTTTAACTTATTATTTATTTTTTGGATTAAAAAATTTAACCTTACTTTTCATTTTATTTTTAATAACATTAATCGTTATAATTCGTGGAACATATGTTCGAATACGATATGATCAACTAATAATAATAGCATGAAAAATAATTCTCCCTCAGAGAATTATTTTTTTATTTTTATCTTACTTCATTTTTCTAATCCTAAACAACTTTATTTTACCTACACTATTTGAGTCAATCTAATATTGAATACTTAAATTCATATAAAAGACCTAACATAATTAGTAAATTAATAAATACAAAAGAAAACATGAACATTAAGCTCTTAGTTATAATTA

TTAAAGGAAAAGGTAAAATTACAACAATTTCAACGTCAAAAATTAAAAAAACAATACCAACAAAAAAAAATTTTAAAGAAAATGGAACCCGAGAAAGCGAAAAAGGATCAAACCCACATTCGAAAGGAGAATTTTTTTCTTTGGCTTCTTTCCCTTGAAAAGCCAAAGAAAAAAATAGACTTATTAATAAAACGATTACCAATAAAATAGTTACATATAAATAAAAAATTATTTAATTTCTTTAAGAAACTATTTAATTGGAAATTAAATGTACTTTTTATACTAATTAAATAATAAATTCATCAATATATAAATGTAAATAAAAATAATCATACTACATCAACAAAATGTCAATATCAGGCTGATGCTTCAAATCCAAAGAAATGATCTGATGAAATAAGTTGATTTTTAATTCGGATATAAGATACTAAAAGAAAAATAGATCCAATAAGGACGTGTAAACCGTGGAAACCTGTAGTTATAAAAAAGGTTGATCCAAAAATACCATCTGAAATTCTAAATTGTGCTTGAAAGTACTCGAAACCTTGGAATATTGTAAAAGCAGCCCCTAATAAAATAGTAATTAATAATGAATTTAAAGCTGATAAGTAATTTTGGTTATAAATTAATAAAGTTAATGAAGTTTTTTATTGAAGTAATTTCTAAAGAGATAGGCATAAATCTATGATTTGTCCCACAAATTTCTGAACATTGGCCATAATATAACCCAGGACGTCTTGAAATTGAAAAAGATTGATTTAAACGGCCAGGAACTGCATCTATTTTAATTCCTAATGATGGAATTGTTCATGAATGAATTACATCTATTGATGAAATTAAATATTTAATGTTAGTATTAATTGGGATTACTAAATTATTGTCGGTATCTAAAAGTCGAAATGAGTTTTTTATTATTTCTGATTCTGGGATTATGAATGAATCAAATTCTTTATTAAAGTCTGAATATTCATAAGATCAATATCATTGATGGCCAAGAATTTTAATAGAAATTTGAGAATTGAATGTTTCATCTGTTAAATACAGTAAATGTAATGAAGGAATAGCAATAAAAATTAATGTAATTGCTGGAATAATTGTTCAAATAATTTCAATTTCTTGGCCTTCTATTATTGATCGTGAAAGTAAATTATTTATTATGATGTTAGTAATTATGTAAATTGTAAGGATAGTAATTATTAAAATAATTATTATTGAATGATCATGAAAAAATACTATTTGCTCTATAATAGGTGAATTTATATCAGAAAAAGATATTTGAGATCAGGTTATCATTAGTTTACTTAAGAATAATATTATTTTGATTGAAAGAGTGTTCTGATGGTGGAAAATTTAATATTCATTCAATAGAAGAATTATTAAATAAGGGGAAATTAATTATTTTTTTTTCAATAATTCTAATTCAAATAATAATAATTAATATAATTACTCCTATTAAGGAGATAATAGATCCTAAGGAAGACACGAAATTTCATTTGGCAAAAAAATCAGGATAGTCTGAGTAACGACGTGGTATACCAGCTAAACCTAAAAAATGTTGTGGGAAAAAGGTTAAATTTACTCCAATAAATGTTACTATAAATTGAACTTTTGTTAACCTTGAGTTTAAGTTTAACCCAAAAAATATTGGAAATCAGTGAATGATAGCTCCTATAATAGCAAATACTGCTCCTATTGATAATACATAATGGAAATGAGCTACAACATAATAAGTATCATGAAGAACAATGTCAATTGAAGAATTGGCTAATATAATCCCAGTAAGTCCTCCTACTGTAAATAGAAAAACAAAGCCTAAAGCTCATAAAATTGAAGTATTAAATTTGATATTAGATCCATGTAAAGTAGCTAGTCAACTAAAAATTTTAATTCCAGTAGGAACTGCAATAATTATTGTTGCTGATGTAAAATAAGCCCGAGTATCTACATCTATACCAACTGTGAATATGTGATGAGCTCATACAATAAATCCTAGTAATCCAATTGCCGCTATAGCATAGATTATTCCTAAATTACCAAAAGGTTCCTTTTTTCCAGTATTATAACAAATGATTTGAGAAAT

CATTCCAAATCCTGGTAAAATTAAAATGTATACTTCAGGATGACCAAAGAATCAAAATAAATGTTGGTATAAAATTGGATCACCCCCTCCTGAAGGATCAAAAAATGAAGTGTTGAAATTTCGATCAGTTAATAATATTGTAATGGCTCCTGCTAAAACAGGCAAAGATAAAAGTAACAAAATAGCAGTAATTAAAACAGATCAAACAAATAGTGGCATTCGTTCTATTGTTATTCCAATAGATCGTATATTTATAATAGTCGTAATAAAATTGATTGCACCTAAAATTGAAGAAGCACCAGCAAGATGAAGAGAGAAAATAGCTAAATCTACTGATGGTCCATAGTGTGATAAATTTGATGATAAAGGCGGATAAACAGTTCATCCAGTTCCTGCTCCTGATTCAATTAATGAGGAATTAATTAATAGAAATAATGAAGGAGGAAGTAATCAAAATCTTATGTTATTTATTCGTGGAAATGCTATATCTGGAGCTCCTAATATAATAGGTACTAATCAGTTTCCAAATCCACCAATTATAATTGGTATTACTATGAAAAAAATTATAATAAATGCATGGGCTGTTACAATTACATTATAAATTTGATCATTTCCAATTAAAGTTCCAGGTTGGCCTAATTCTATTCGAATTAATATTCTTATTCTTAGTCCTAATATTCCTGATCATGCCCCAAAAATTAAATATATTGTTCCAATGTCTTTATGATTAGTAGAATATATTCATCGCGGTAAAATGGCTGAAGTTTAGGCGATAAATTGTAAATTTATTTATGAATAATTTCTTTTACTATTAAGATTTATTAATAATAATTTTTACTTTGAAGGCAAATAGTTTTTTTAACTTAAAATCTTTTTAAAAAAAAATATTAACTAAAATAATAAGAATAATTGTATTAATTTTAATTAAAATATTTCTTTTCATTATTTGAAGGTTTCATCTTAAAAATTTTAAATTAGTGAAAAAAAATGGGGTTAAAATGCGTAAATAAAAAAATAAGTTAATTAATGAAGAAATAATTAAGATAATTATAAAAATTAAATTTATTTTAATTAAGTATAAAATAGCTATAATTTTTATAAAGAAACCAAGAAAGGGGGGTATTCCAGCTAAAGATATTATTGATATGATTATAGTATTTTTTATTTTGATTCTTATTTTGTTGCTTATTAAATTGTTTAATCTATTAAAATTAACTGCTTTACAATTAGTAATAATAGTATAAATAATAGTTGAGTATATAATTAAATATGAAATTCAAAAATTCATTTTTTTAGCAATTAAACATAAAATTCATCCTTGATGGGAAATTGAAGAAAGAATTAAAATTTTTTTTATTAATTTGTGTTTTATTGCTATAATAGAAGCTATTAGTGAGGATAAAACAAATAAAGGAATTATTGAATTATTTATAAATTTTTCAATAATTAATAAGGGAATAATTTTTTGAATAGTTAGTAAAATAAGAAGTGAATAAAAAGTTAATGACTCTCTAATTAAGATTAATCAAAAATGAAACGGAATTATTCCTAATTTAATTAAAATTGAAATATTAATTAGATTGAAAAATAATCTCATATTAAATAATCTATATTGAAAAGAGGAGATAAAGAAGAGGGAAGAAGAAAAAGATTGAATAATAAAATATGTGATTATAGAATTATAATTTTTTAATTTGAATTCATTTATGATTGGAATAAAAGATATTATGTTAATTTCTATTATTAATCAAAAAATAAATCATGAATTTGATGATAAAGAAGTTAAAATTGAAATAATAATAACTCAAAGTATTATTTTTTTAAAAAAAATTAATAAAGGATTTTGTAATCATATTTGGGGTATGAACCCAATAGCTTAATTTAGCTTACTTTATTTAAAATCTTAGATAAATATAATTTATCAATTTTTAAATTTGCAGTTTAATTTCAATAAGATTTTTGCATGCAATGTATTATCTCATCTATTGTAAATAAGGGGCGACGACCGGCTGTCTCATCTATTGACCCATAGGAGAGTAACAAAGGGTCATACTAAGTCATGTACACATCTACTAATTAATAAATTCGGAGAAAAAAAGGGAGATGGTTACATTTTGCTCAAATTATAGGACATATTTTCAAATA


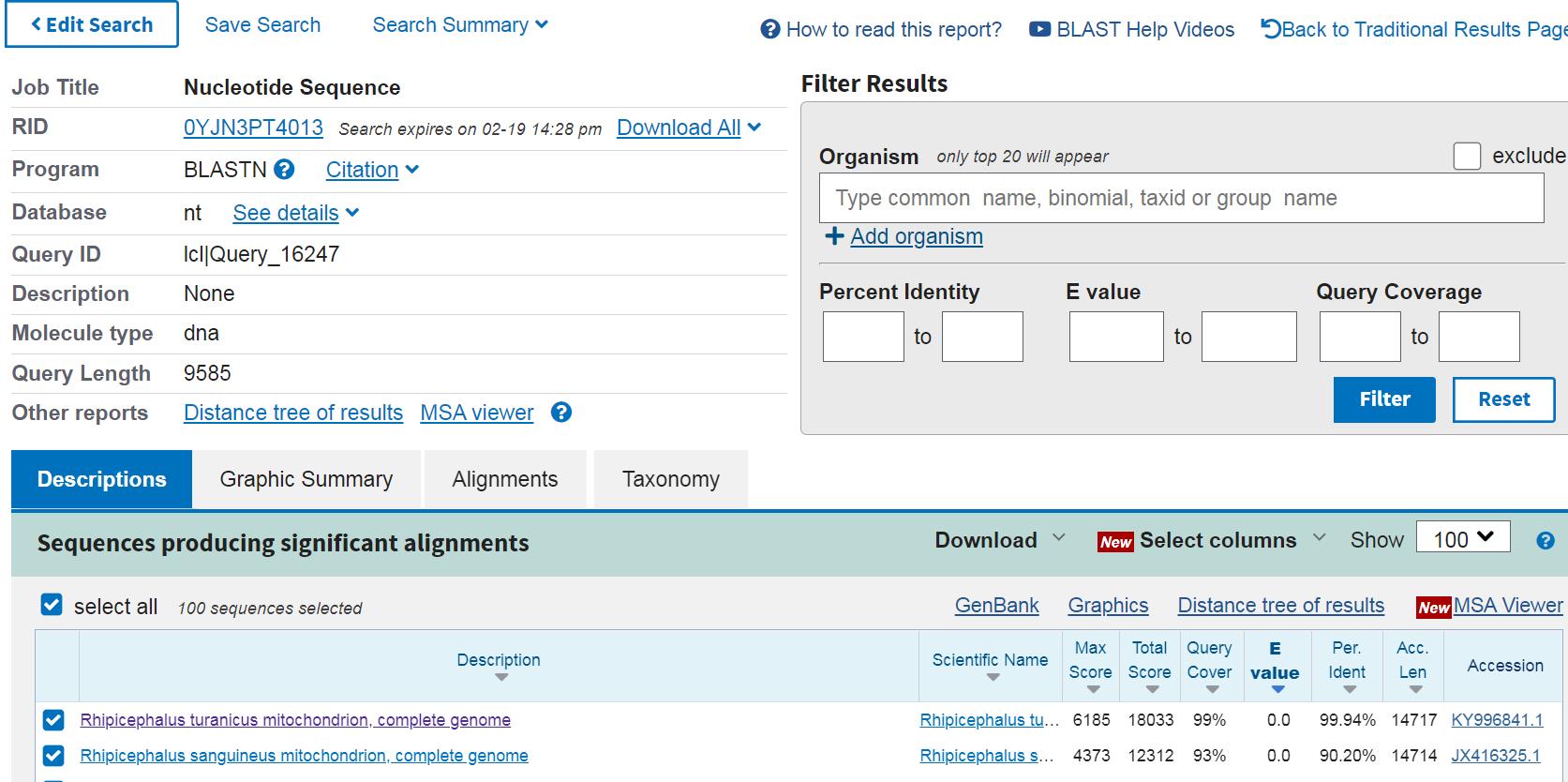

Supplement: Supplementary file 1 — Additional file 1. The partial mitochondrial genome (9585 bp) used to identify species and the BLAST results against NCBI. [file 13071_2022_5214_MOESM1_ESM.docx]
