# Supplementary figures and images for: Developmental transcriptomics throughout the embryonic developmental process of Rhipicephalus turanicus reveals stage-specific gene expression profiles
Source: Parasit Vectors. 2022 Mar 15;15:89. doi: 10.1186/s13071-022-05214-w (PMC8922761; doi:10.1186/s13071-022-05214-w)

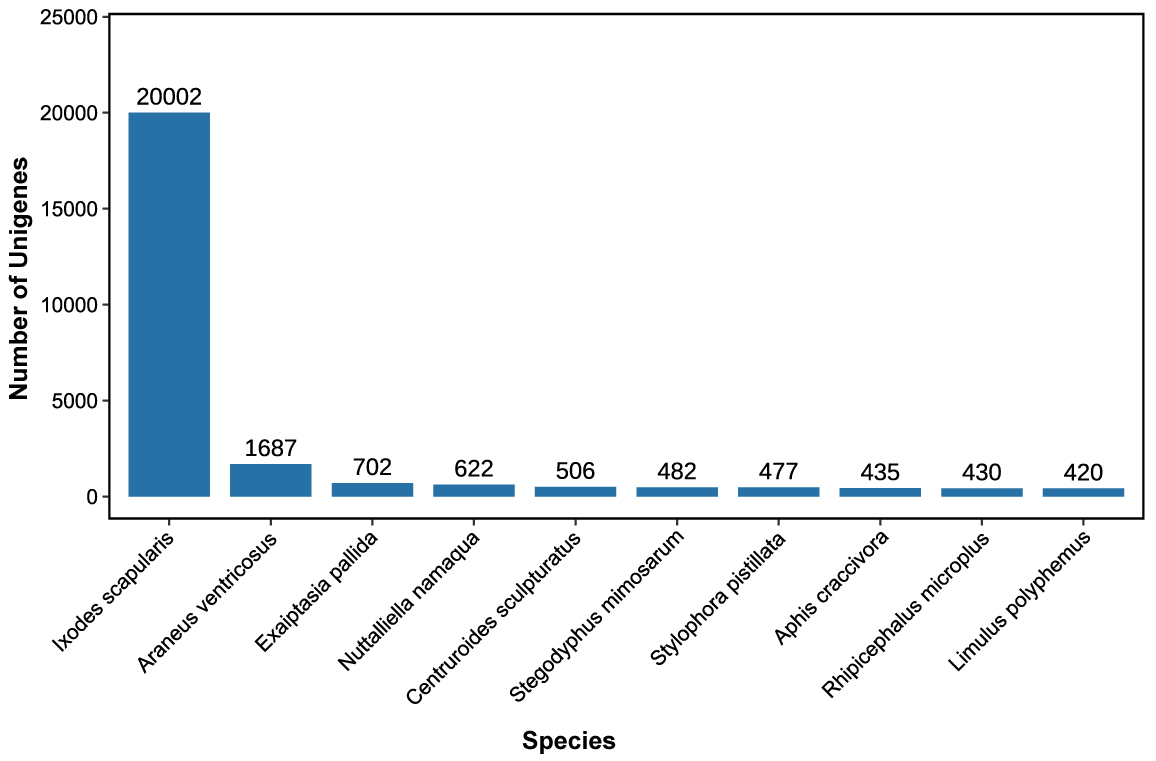

Supplement: Supplementary file 2 — Additional file 2: Figure S1. Species distribution of Nr annotation results. The x-axis represents the name of species; the y-axis represents the number of unigenes annotated to different species. [file 13071_2022_5214_MOESM2_ESM.tif]

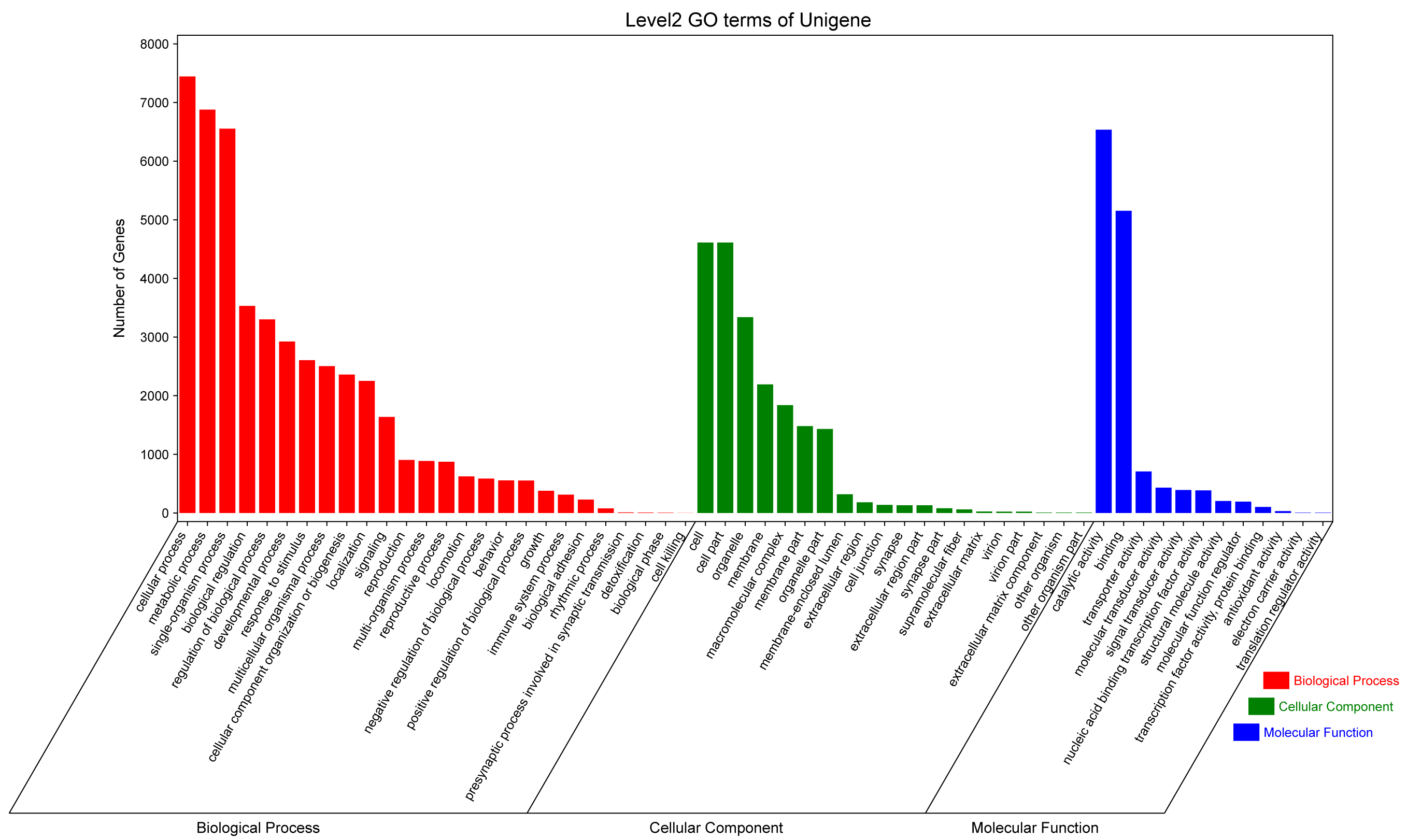

Supplement: Supplementary file 3 — Additional file 3: Figure S2. GO functional classification of unigenes. The x-axis represents the GO term; the y-axis represents the number of genes classified into each GO term. [file 13071_2022_5214_MOESM3_ESM.tif]

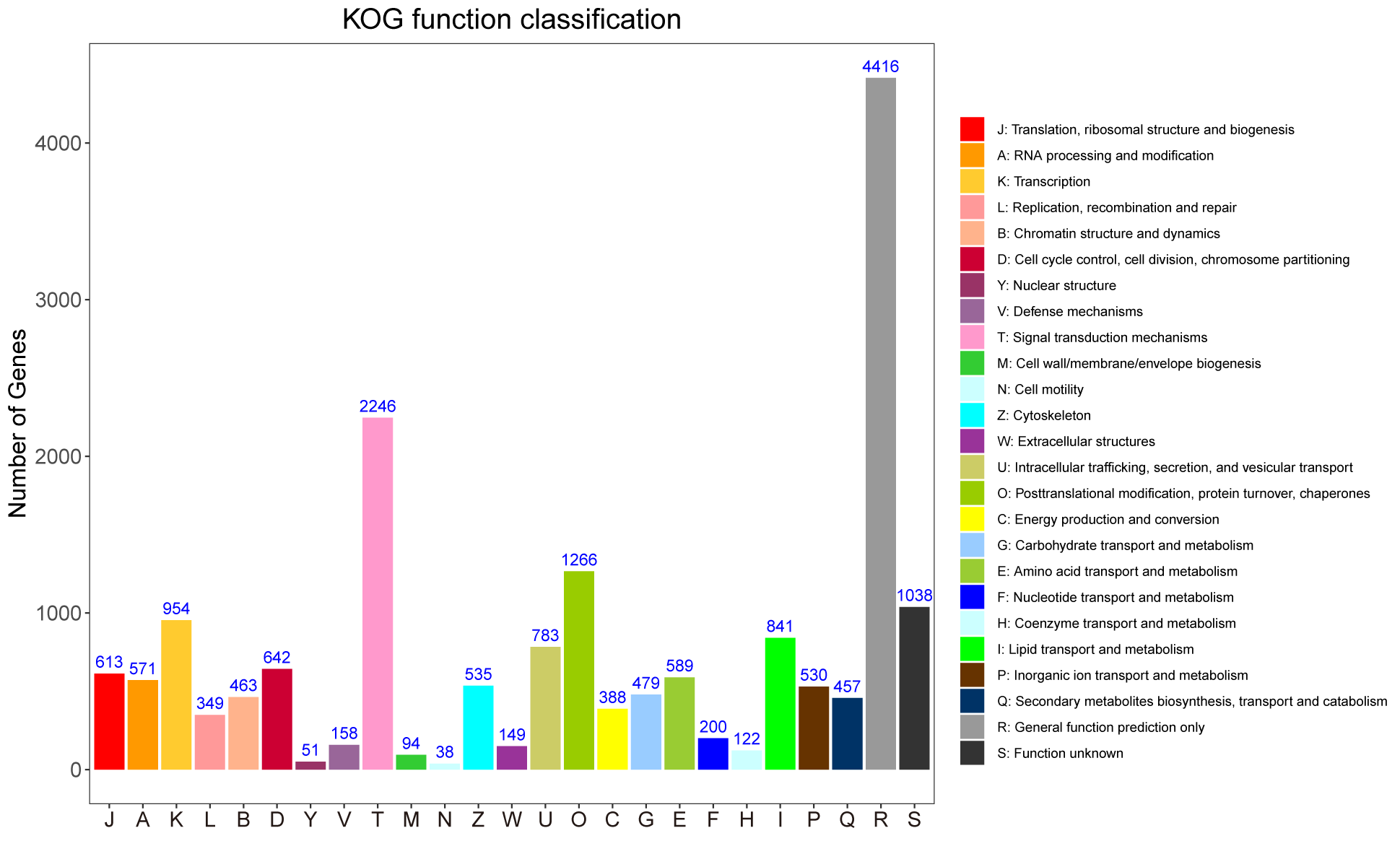

Supplement: Supplementary file 4 — Additional file 4: Figure S3. COG functional classification of unigenes. The x-axis represents the COG category; the y-axis represents the number of genes classified into each COG category. [file 13071_2022_5214_MOESM4_ESM.tif]

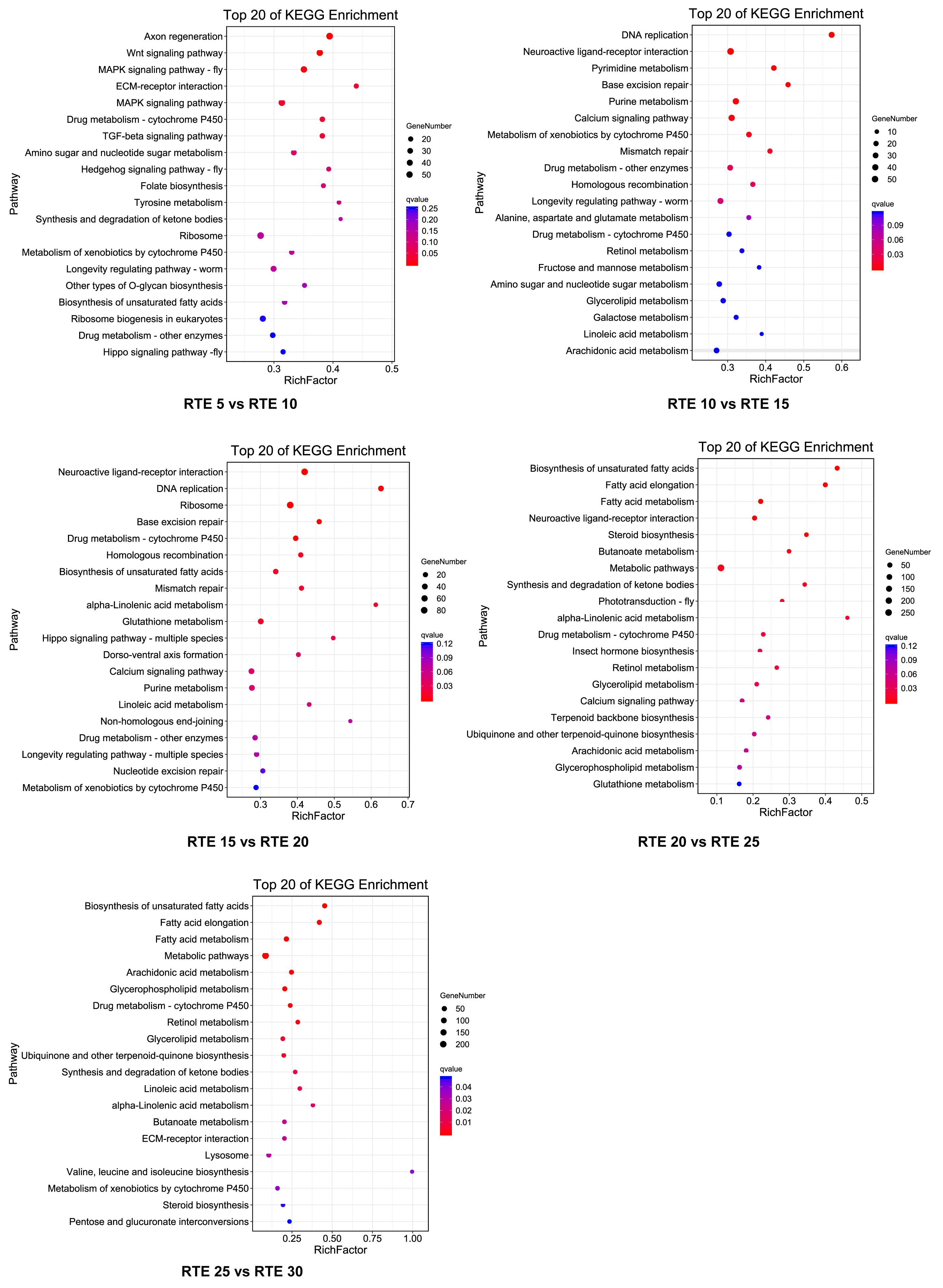

Supplement: Supplementary file 5 — Additional file 5: Figure S4. KEGG enriched pathway analysis of genes between adjacent embryonic developmental groups. The x-axis represents the rich factor, the y-axis represents KEGG pathways. Rich factor refers to the ratio of the number of genes located in the KEGG pathway and the total number of genes in the KEGG pathway. The larger the rich factor, the greater the enrichment. [file 13071_2022_5214_MOESM5_ESM.tif]

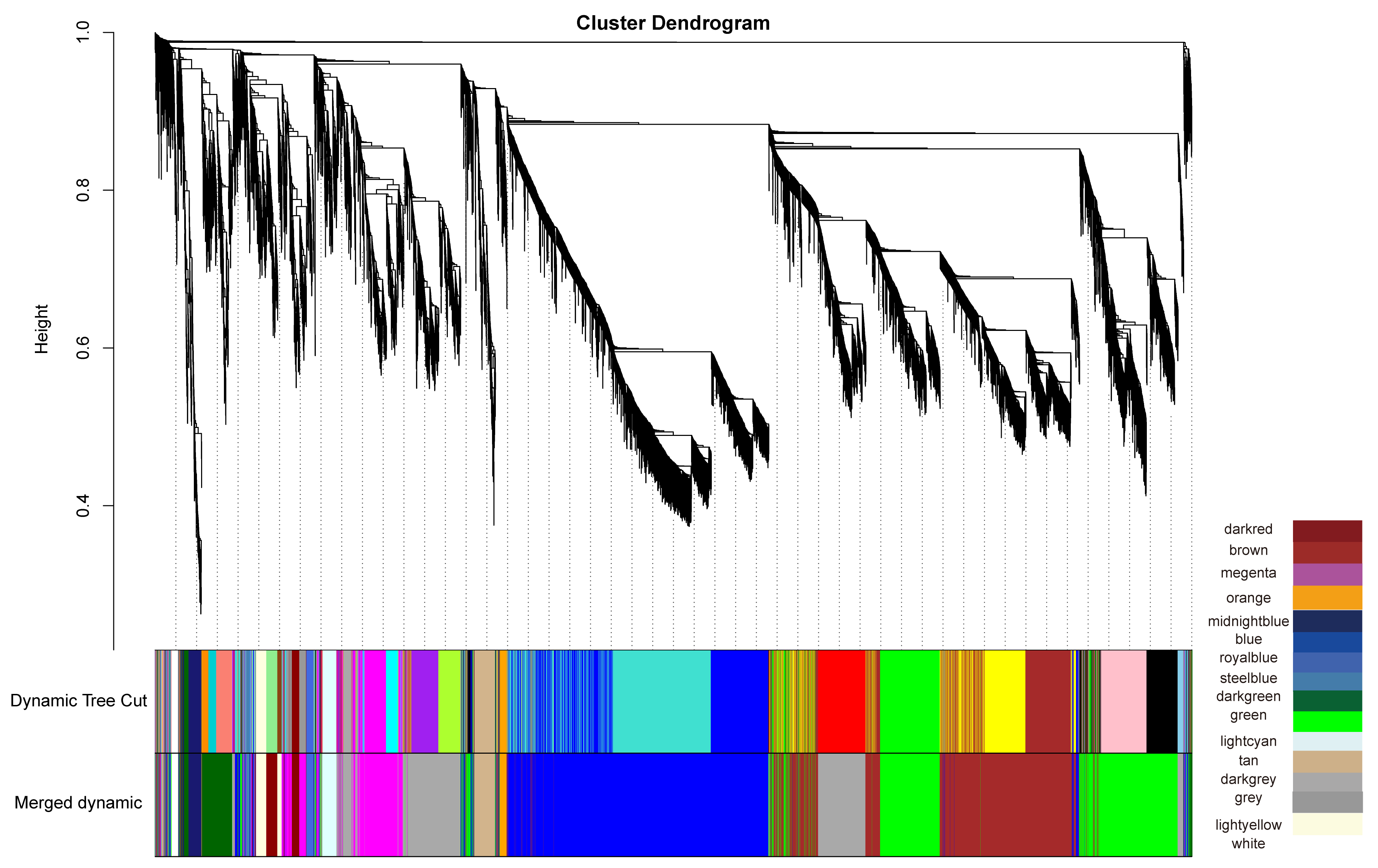

Supplement: Supplementary file 6 — Additional file 6: Figure S5. WGCNA of genes in different embryonic developmental stages. Hierarchical cluster tree showing co-expression modules identified by WGCNA. Each leaf on the tree represents one gene. All genes were grouped into 16 different color-coded modules according to their expression pattern. [file 13071_2022_5214_MOESM6_ESM.tif]

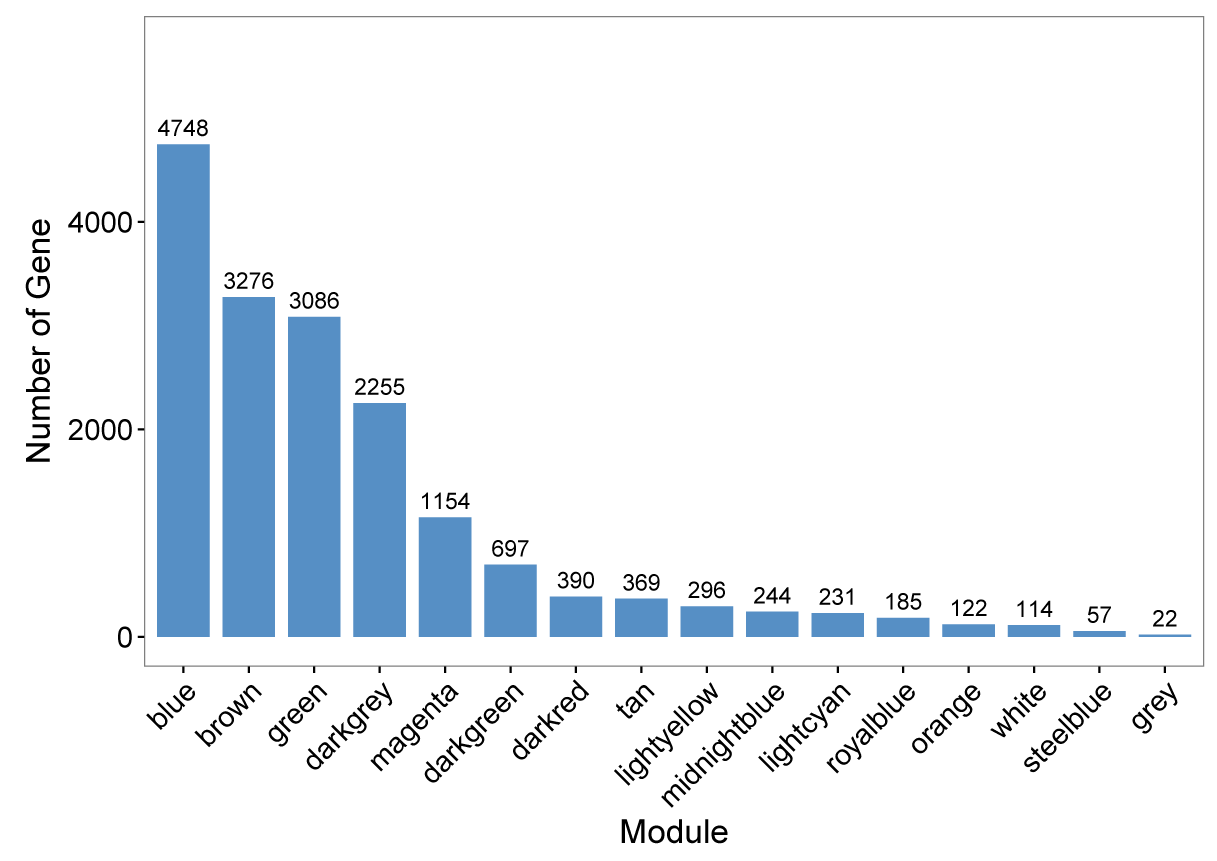

Supplement: Supplementary file 7 — Additional file 7: Figure S6. Gene numbers of different modules. [file 13071_2022_5214_MOESM7_ESM.tif]

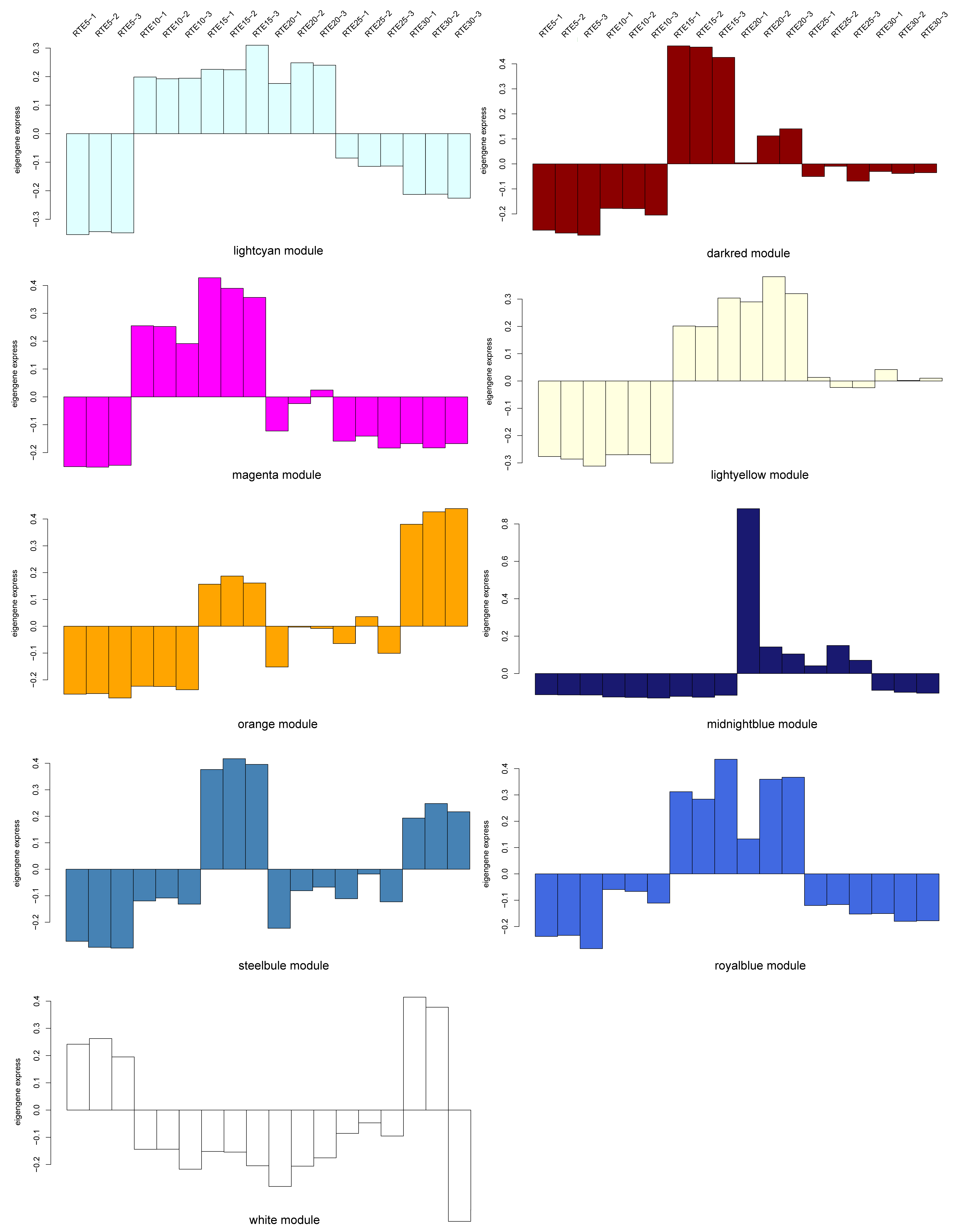

Supplement: Supplementary file 8 — Additional file 8: Figure S7. Express pattern of lightcyan, darkred, magenta, lightyellow, orange, midnight, steelblue, royalblue, and white modules. The x-axis represents the sample; the y-axis represents the expression profile of the eigengene. [file 13071_2022_5214_MOESM8_ESM.tif]

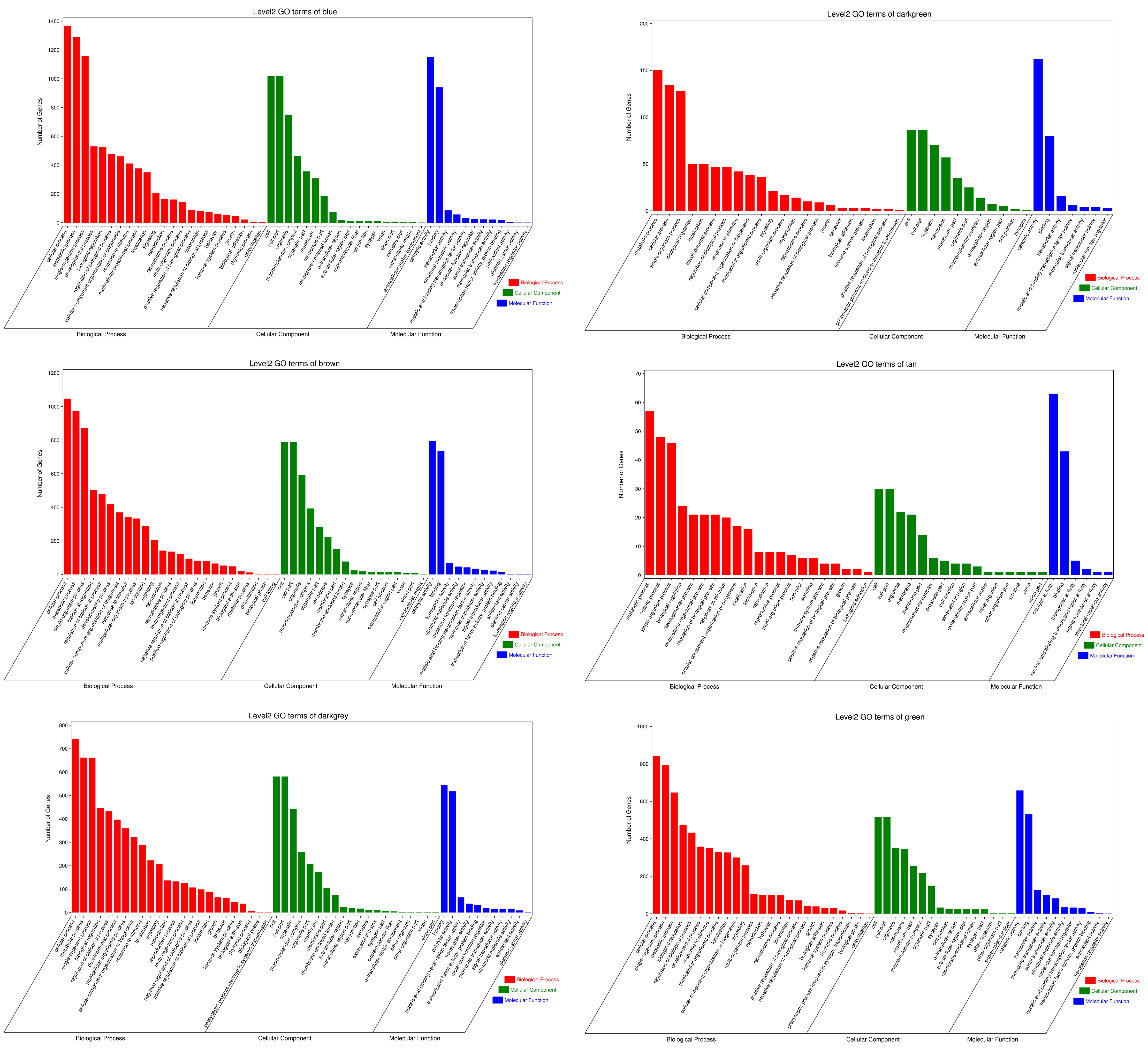

Supplement: Supplementary file 9 — Additional file 9: Figure S8. GO functional classification of the blue, darkgreen, brown, tan, darkgrey, and green modules. The x-axis represents GO terms; the y-axis represents the number of genes classified into each GO term. [file 13071_2022_5214_MOESM9_ESM.tif]
