# Supplementary material for: Developmental transcriptomics throughout the embryonic developmental process of Rhipicephalus turanicus reveals stage-specific gene expression profiles
Source: Parasit Vectors. 2022 Mar 15;15:89. doi: 10.1186/s13071-022-05214-w (PMC8922761; doi:10.1186/s13071-022-05214-w)
Supplement: Supplementary file 10 — Additional file 10: Table S1. Primers used in qRT-PCR. [file 13071_2022_5214_MOESM10_ESM.doc]

Table S1. Primers of eight genes used for qRT-PCR

| Gene name |  | Primers | |  |
| --- | --- | --- | --- | --- |
| unigene0032012/ Nop60B |  |  | F: GCGTCAGGTCAGGCATTCA | |
|  |  |  | R: TACTCCTCGTCTCGGTGGTT | |
| unigen0086511 |  |  | F: GACACCTCAGCCTCCACAA | |
|  |  |  | R: TGCTCAATCATCTCTGCTTCTC | |
| unigene0101177/SRSF7 |  |  | F: TTGCTACGACTGCGGTGAA | |
|  |  |  | R: AGAGAACGAACGGCTCCTG | |
| unigene0070965/HSP90AA1 |  |  | F: CAGTTACATTACACGGCTTGGA | |
|  |  |  | R: CGCTGTTCGCTCTGACTTG | |
| unigene0039241/NOP58 |  |  | F: TTGACTTGTGCTGGTGTATCG | |
|  |  |  | R: AAGAAGAGGAGGAGGAAGAAGG | |
| unigene0071552/rbm4.1 |  |  | F: TCCGTCGTAGCCGTTCCTT | |
|  |  |  | R: CACAAGCAAGTTCCACCAAGAG | |
| unigene0103757/UBA1 |  |  | F: CGGCGTGTCTCAAGGATGT | |
|  |  |  | R: GTGGCGATGGCAGGTATGA | |
| unigene0004692/CAM |  |  | F: CTGTGGAGGATCGGAAGTCTAA | |
|  |  |  | R: CTGGTGTGAGGTGGTGAAGT | |
